# In Silico Molecular Docking and Pharmacokinetic Evaluation of Cannabinoid Derivatives as Multi-Target Inhibitors for EGFR, VEGFR-1, and VEGFR-2 Proteins

**DOI:** 10.3390/cimb48020204

**Published:** 2026-02-12

**Authors:** Akhtar Ayoobi, Hyong Woo Choi

**Affiliations:** 1 Department of Plant Medicals, Gyeongkuk National University, Andong 36729, Republic of Korea; akhtar.ayoobi@gmail.com; 2 Institute of Cannabis Biotechnology, Gyeongkuk National University, Andong 36729, Republic of Korea

**Keywords:** cannabinoids, EGFR, VEGFR-1, VEGFR-2, molecular docking, ADME, pharmacokinetics

## Abstract

Cancer therapy development increasingly focuses on multi-target approaches to inhibit key proteins involved in tumor growth and angiogenesis. This study explored the potential inhibitory interactions of 110 cannabinoid derivatives using molecular docking simulations against epidermal growth factor receptor (EGFR), vascular endothelial growth factor receptor-1 (VEGFR-1), and VEGFR-2. Blind docking with AutoDock Vina identified eight recurrent hits across all three targets, including polar THC glucuronides and more drug-like cannabinoid scaffolds. Among these, 2′-Hydroxy-Delta (9)-THC and Ajulemic Acid combined favorable multi-target binding with superior predicted pharmacokinetic properties compared with other cannabinoids and reference inhibitors (lapatinib, motesanib, and sorafenib). ADME predictions highlighted Ajulemic Acid as the most promising oral candidate, showing optimal molecular weight, high oral bioavailability, and good gastrointestinal absorption, while 2′-Hydroxy-Delta (9)-THC exhibited potential for central nervous system exposure due to predicted blood–brain barrier permeability. In contrast, glucuronidated THC metabolites and highly lipophilic cannabinol esters displayed strong docking scores but suboptimal drug-likeness, suggesting prodrug- or metabolite-like behavior rather than suitability as primary oral leads. Toxicity predictions classified all compounds as moderately toxic, with Ajulemic Acid showing a comparatively more favorable safety profile. These findings do not demonstrate biological inhibition and should be interpreted strictly as hypothesis-generating computational evidence, providing a rational framework for future in vivo and in vitro validations.

## 1. Introduction

Plant-derived compounds have long served as important sources of pharmacologically active scaffolds in drug discovery, contributing to the development of numerous therapeutic agents [1]. Many clinically used drugs originate from natural products, underscoring the continued relevance of plants as reservoirs of bioactive chemical diversity [2]. *Cannabis sativa* L. has attracted considerable scientific interest due to its diverse phytochemical composition and associated therapeutic properties [3]. The plant produces a wide range of secondary metabolites, most notably structurally diverse cannabinoids characterized by a terpenophenolic scaffold, which form the basis of its biological activity. Major cannabinoids arise from shared biosynthetic pathways and can be converted from acidic precursors into pharmacologically active neutral forms such as cannabidiol (CBD) and tetrahydrocannabinol (THC) [4,5]. Cannabis plants exhibit a broad spectrum of biological activities, with reported effects on sleep regulation [6], inflammation [7], neurodegeneration [8], pain [9], nausea [10], and appetite loss [11]. In addition to THC, cannabis contains multiple bioactive cannabinoids such as cannabidiol (CBD) and cannabinol (CBN), together with numerous terpenes and phenolic compounds, whose structural diversity underlies their distinct biological effects and non-psychoactive profiles [5,12,13]. This chemical diversity provides a rationale for investigating cannabinoids using in silico and in vitro approaches to explore their potential relevance in cancer-related pathways [14,15]. Cannabinoids such as CBD, CBN, and cannabigerol (CBG) have been identified as potential candidates for targeting various cancers, including breast, lung, and brain cancers, as well as leukemia, melanoma, and myeloma [16]. CBD has been reported to modulate cancer-related cellular processes in head and neck squamous cell carcinoma (HNSCC), including effects on cell migration, invasion, and viability, primarily in preclinical models [17]. Among Cannabinoids, only THC and CBD have advanced to clinical trials for cancers like glioma, neuroblastoma, and leukemia, with most other cannabinoids still at the in vivo testing stage due to observed side effects in animal models. This gap underscores the need for further research into the bioactivity and therapeutic potential of these cannabinoids [16,18].

Cancer remains a leading cause of mortality globally, despite advancements in therapeutic approaches such as hormonal therapy, radiotherapy, surgery, and targeted molecular therapies [19]. Given the critical role of Epidermal Growth Factor Receptor (EGFR) tyrosine kinase in regulating cell survival and proliferation, its inhibition through tyrosine kinase inhibitors (TKIs) has shown particular promise for different cancers such as lung, breast, and colorectal cancers [20,21]. EGFR overexpression, commonly observed in aggressive cancers, further underscores the significance of TKIs in oncology [22]. Although FDA-approved EGFR-TKIs like gefitinib, erlotinib, and osimertinib have transformed treatment paradigms for cancers such as non-small cell lung cancer (NSCLC), challenges related to drug resistance and toxicity persist, emphasizing the need for novel therapeutic approaches [23]. Recent research into phytochemicals, such as curcumin and resveratrol, suggests their potential as effective EGFR inhibitors, complementing or enhancing traditional TKIs while reducing toxicity [24,25]. In this context, cannabinoids, such as CBD, CBG, and CBN, are emerging as potential EGFR-targeted therapies in cancers like breast and lung [26], suggesting a potential rationale for exploring cannabinoid interactions with EGFR at the molecular level.

In addition to EGFR inhibition, targeting angiogenesis has become another cornerstone of cancer therapy. Angiogenesis, essential for processes such as embryonic development and wound healing, is tightly regulated by pro- and anti-angiogenic signals [27,28]. Judah Folkman’s theory on the necessity of angiogenesis for tumor progression laid the foundation for anti-angiogenic therapy [29], which aims to limit tumor growth by restricting its vascular supply. Vascular Endothelial Growth Factors (VEGFs) and their receptors (VEGFRs) are primary drivers of tumor angiogenesis [30]. VEGFR-1 activation promotes tumor cell invasion, chemotaxis, and immune evasion through interactions with myeloid progenitors and tumor-associated macrophages. Targeting VEGFR-1-specific ligands, such as VEGF-B and PlGF, along with selectively blocking VEGFR-1 activation, presents a potential strategy to inhibit tumor-associated angiogenesis and malignancies independent of blood vessel formation. Furthermore, suppressing VEGFR-1 signaling decreases tumor cell survival and invasiveness, hinders myeloid progenitor mobilization, and prevents infiltration of protumoral M2 macrophages into tumors [30]. VEGFR-2 activation initiates signaling pathways such as PI3K-AKT-mTOR and PLC-ERK1/2 that promote endothelial cell proliferation and migration, both of which are essential for tumor vascularization [31,32]. The overexpression of VEGFR-2 in solid tumors, including breast cancer and hepatocellular carcinoma, underscores its significance as a therapeutic target [33,34]. However, the emergence of resistance and adverse effects such as hypertension and gastrointestinal toxicity limit the use of VEGFR-2 inhibitors [35]. Consequently, there is an urgent need for novel VEGFR-1 and VEGFR-2 inhibitors with improved efficacy and reduced side effects.

Molecular docking is a pivotal technique in drug discovery, enabling researchers to simulate the interaction between small molecules and target proteins, thereby predicting binding affinities and the potential efficacy of drug candidates. This method facilitates the virtual screening of extensive compound libraries, streamlining the identification of promising therapeutic molecules prior to experimental validation [36]; for instance, a prior study utilized structure-based bioinformatics and virtual screening to identify a promising fibroblast growth factor receptor 2 (FGFR2) inhibitor, as a therapeutic target, given its role in various cellular processes and its implication in diseases such as cancer [37]. In this method, by analyzing the binding interactions at specific protein sites, molecular docking not only aids in identifying active compounds but also informs the design of inhibitors targeting key proteins in diseases [38,39]. Consistently, recent in silico investigations have applied molecular docking and integrated computational pipelines to prioritize plant-derived neuroactive compounds and assess their drug-likeness, blood–brain barrier permeability, and multi-target engagement in complex neurological disorders [40,41]. Advances in docking algorithms continue to enhance the accuracy and efficiency of this approach, solidifying its role as an essential tool in modern drug development [42].

In this study, we developed a curated library of 110 cannabinoids and employed molecular docking as a proof-of-concept computational framework to explore their interactions with three key cancer-related targets, EGFR, VEGFR-1, and VEGFR-2. The specific objectives of this work were:

(1) to perform a systematic in silico virtual screening of cannabinoid compounds in order to evaluate their ability to engage the active regions of these clinically relevant proteins; (2) to analyze binding affinities and interaction patterns to prioritize cannabinoid scaffolds exhibiting consistent and selective multi-target engagement; and (3) to assess the drug-likeness and pharmacokinetic feasibility of the prioritized scaffolds, thereby refining computational hits toward biologically relevant candidate molecules. Rather than providing definitive evidence of therapeutic efficacy, this study establishes molecular docking as a rational first step for integrating plant-derived cannabinoids into more advanced cancer drug discovery pipelines and for guiding subsequent in vitro and in vivo validation.

## 2. Materials and Methods

### 2.1. Ligand Preparation

A comprehensive library of 110 cannabinoid derivatives was assembled as ligands, sourced from the PubChem Compound and Substance Database (https://pubchem.ncbi.nlm.nih.gov/, accessed on 21 January 2026). These compounds (detailed in Supplementary Table S1) were initially obtained in SDF format and subsequently processed for molecular docking studies. In addition, well-established positive controls were incorporated into the docking study: Lapatinib (PubChem CID: 208908, C29H26ClFN4O4S) for EGFR, Motesanib (CID: 11667893, C22H23N5O) for VEGFR-1, and Sorafenib (CID: 216239, C21H16ClF3N4O3) for VEGFR-2. Docking was performed using the PubChem-deposited stereoisomer for each compound. These ligands were selected based on their strong binding affinities and specific inhibitory effects on their respective targets, providing reliable benchmarks for evaluating the effectiveness of the cannabinoid derivatives. All cannabinoid derivatives and positive control ligands were energy-minimized using the Open Babel minimizer implemented in PyRx (Python Prescription 0.8), while AutoDockTools (version 1.5.7, The Scripps Research Institute, La Jolla, CA, USA) was used solely for hydrogen addition, charge assignment, and PDBQT conversion [43]. Energy minimization was performed using a classical force-field–based approach, which is widely accepted and computationally efficient for large-scale docking and virtual screening studies. Ab initio optimization methods are typically reserved for small ligand sets or molecular dynamics parameterization and were therefore not applied in this screening-oriented workflow.

### 2.2. Target Protein Optimization

The crystal structures of the target proteins, EGFR, VEGFR-1 and VEGFR-2, were obtained from the Protein Data Bank (PDB) (http://www.rcsb.org/, accessed on 21 January 2026). These structures were retrieved in PDB format (.pdb) using the following IDs: EGFR (1M17), VEGFR-1 (3HNG) and VEGFR-2 (3U6J). The selected protein structures were chosen based on structural resolution, functional relevance, and suitability for molecular docking. All PDB entries used exhibit high or moderate resolution, providing reliable atomic coordinates for ligand–protein interaction analysis. Where available, structures containing co-crystallized inhibitors were selected to support accurate identification of active binding regions and biologically relevant docking poses. Although more recent kinase structures exist, the selected PDB entries represent well-characterized conformational states that are widely used in structure-based drug discovery and are appropriate for proof-of-concept virtual screening. Prior to docking, protein structures were prepared following a standard AutoDock-based workflow, including removal of crystallographic water molecules and co-crystallized ligands, correction of missing atoms, addition of polar hydrogens, and charge assignment. No additional global molecular mechanics energy minimization of the protein structures was applied. Docking simulations were performed using the resulting ligand-free protein structures, which were saved in PDBQT format for docking compatibility [43]. This approach was adopted given the use of experimentally resolved crystal structures and the screening-oriented aim of the study, which focused on comparing relative binding trends across compounds rather than predicting absolute binding free energies. The potential impact of protein rigidity on docking accuracy is therefore considered a limitation of this analysis.

### 2.3. Active Site Prediction

**Figure 1:**
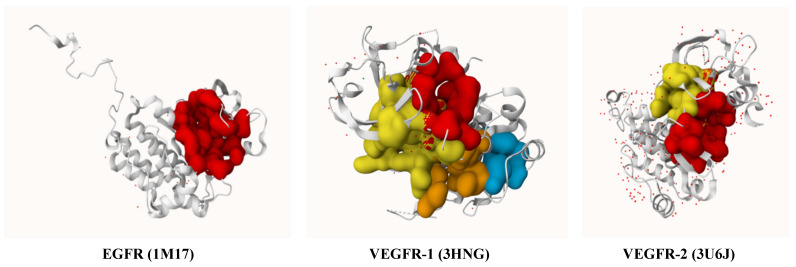
3D visualization of the active site pockets in EGFR (1M17), VEGFR-1 (3HNG), and VEGFR-2 (3U6J) as predicted by PrankWeb.

The active sites of target proteins were identified using the PrankWeb web server (https://prankweb.cz/, accessed on 21 January 2026), a reliable tool for analyzing and predicting potential binding sites on protein structures [44]. The predicted active sites for each target protein are summarized in Supplementary Table S2, and their corresponding binding pockets are illustrated in Figure 1, with highlighted regions as specified in Supplementary Table S2. These binding sites provided a refined focus for docking simulations, ensuring that interactions occurred in biologically relevant areas.

### 2.4. In Silico Molecular Docking and Visualization of Interactions

Docking simulations were performed using AutoDock Vina implemented in the PyRx virtual screening platform [45]. Binding affinities of the 110 cannabinoid derivatives were initially ranked based on the lowest predicted binding energy scores. A blind docking strategy was employed by defining a grid box encompassing the entire protein surface, allowing unbiased exploration of all potential ligand-binding sites. Although kinase ATP-binding pockets are well characterized, blind docking was intentionally applied to evaluate whether cannabinoids preferentially localize within the canonical ATP-binding cleft or exhibit affinity toward alternative or secondary binding regions.

For each ligand, multiple docking poses generated by AutoDock Vina were examined and compared in terms of binding location, orientation, and interaction pattern. The selected top-ranked conformation consistently belonged to the dominant and most representative binding mode, characterized by highly similar poses clustered within the same binding pocket and exhibiting comparable interaction profiles. No alternative high-scoring clusters corresponding to distinct binding sites or substantially different binding modes were observed for the prioritized ligands. Molecular interaction analyses were visualized using BIOVIA Discovery Studio Visualizer (v24.1.0.23298) [46] for two-dimensional representations and UCSF ChimeraX (version 1.7.1) [47] for three-dimensional visualization, enabling detailed assessment of ligand–protein interaction patterns. Pre-docking molecular dynamics simulations were not conducted, as this study focused on docking-based virtual screening and scaffold prioritization. MD-based conformational refinement is generally applied in later-stage, ligand-specific optimization studies and was therefore considered beyond the scope of the present analysis.

### 2.5. ADME and Toxicity Predictions

#### 2.5.1. ADME Analysis

The ADME (Absorption, Distribution, Metabolism, and Excretion) profiles of the cannabinoid derivatives were analyzed using SwissADME (http://www.swissadme.ch/, accessed on 21 January 2026) [48]. This tool evaluated pharmacokinetics, drug-likeness, and molecular properties, including gastrointestinal absorption, blood–brain barrier permeability, and potential drug interactions. The analysis provided preliminary insights into the therapeutic viability of the compounds, streamlining the selection of candidates for experimental validation.

#### 2.5.2. Toxicity Prediction

Toxicity profiles of the cannabinoid derivatives were evaluated using the ProTox-3 platform (https://tox.charite.de/protox3/, accessed on 21 January 2026) [49]. Parameters such as LD_50_, hepatotoxicity, cytotoxicity, and immunotoxicity were predicted, enabling a systematic assessment of safety and tolerability. This computational approach facilitated the selection of safe and promising compounds for further studies.

## 3. Results

### 3.1. In Silico Molecular Docking

Molecular docking studies were conducted using AutoDock Vina integrated with PyRx to investigate the binding interactions of 110 cannabinoid derivatives with three target proteins: EGFR (1M17), VEGFR-1 (3HNG), and VEGFR-2 (3U6J). A blind docking approach was employed to enable ligand binding across the entire protein structure, ensuring the identification of interactions beyond the expected active sites. Specific positive control ligands were chosen for each target to benchmark the docking results against cannabinoid derivatives. For EGFR (1M17), Lapatinib (CID: 208908) [50] was selected as a positive control based on its superior binding affinity, while Motesanib (CID: 11667893) and Sorafenib (CID: 216239) were chosen for VEGFR-1 and VEGFR-2, respectively [51,52] (Table 1, Table 2 and Table 3). Docking simulations generated nine binding conformations for each ligand–protein complex, all of which were inspected in terms of binding affinity, binding location, and interaction patterns. The top-ranked conformation was selected only when it showed a biologically relevant binding mode within the known active site, while alternative poses typically exhibited weaker affinities or unfavorable orientations. The top five compounds for each protein were shortlisted based on binding affinity scores and visualized using BIOVIA Discovery Studio Visualizer and UCSF ChimeraX to gain insights into ligand–protein interactions.

#### 3.1.1. Docking Interaction of EGFR (1M17) Protein with Cannabinoid Derivatives

**Figure 2:**
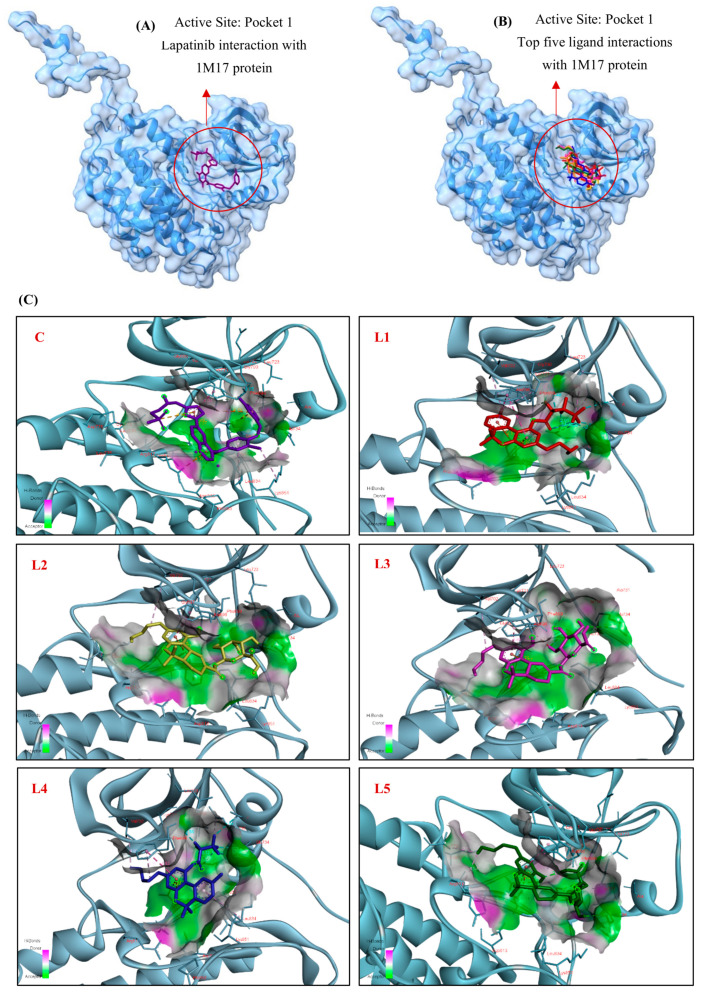

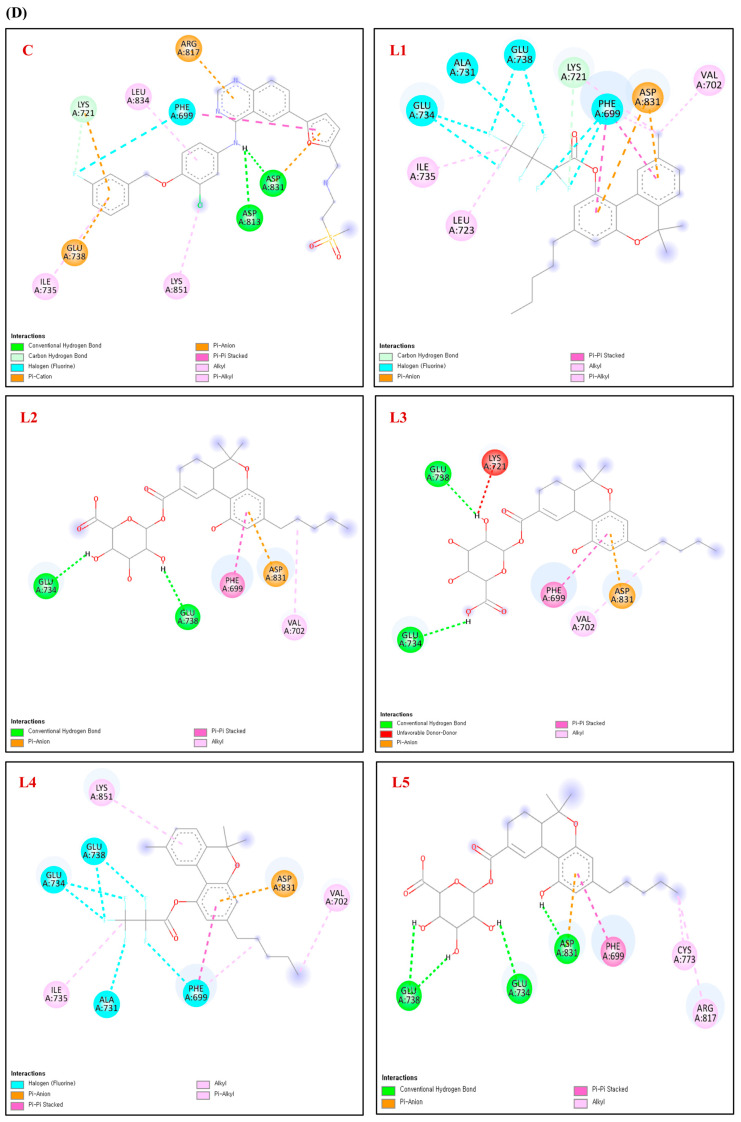
3D Molecular docking simulations of (**A**) Lapatinib and (**B**) the top five cannabinoid derivatives at the active site of EGFR (PDB ID:1M17), visualized using UCSF ChimeraX. (**C**) 3D and (**D**) 2D interaction diagrams depicting the binding poses of Lapatinib and the top five cannabinoid derivatives with EGFR (PDB ID:1M17), generated using BIOVIA Discovery Studio (C: Control, L1: Ligand 1, L2: Ligand 2, L3: Ligand 3, L4: Ligand 4, L5: Ligand 5). The ligands are color-coded: purple represents Lapatinib (C), red for Cannabinol, Heptafluorobutyrate (L1), yellow for 11-Nor-9-Tetrahydro Cannabinol-9-Carboxylate Acyl -D- glucuronide (L2), pink for THC-11-Oic Acid Glucuronide (L3), blue for Cannabinol, Pentafluoropropionate (L4) and green for 11-Nor-9-Carboxy-Delta9-Tetrahydrocannabinol Glucuronide (L5).

To explore potential binding interactions across the EGFR (1M17) protein structure, blind docking simulations were conducted on 110 cannabinoid derivatives, with Lapatinib serving as the positive control. Docking was performed using AutoDock Vina implemented in PyRx. A protein-wide blind docking approach was applied with a grid box centered at (8.22, 7.15, 7.59 Å) and dimensions of 94.3 × 67.4 × 51.5 Å. The exhaustiveness parameter was set to 8. No manual clustering parameters were defined, and poses were ranked based on Vina binding affinity scores. Lapatinib was selected for its well-established mechanism of action, which involves competing with ATP at the ATP-binding domain on the cytoplasmic tail of the tyrosine kinase receptor [41]. The 3D positions of Lapatinib and the top five ligands within the active site of EGFR are illustrated using UCSF ChimeraX (Figure 2A,B). Additionally, 2D and 3D visualizations highlighting key amino acid residues involved in the interactions are presented using Discovery Studio Visualizer (Figure 2C,D). Interestingly, the top five binding results closely aligned with the predicted active site, which also hosted Lapatinib in control docking (Figure 1 and Figure 2A,B). Lapatinib, with a binding affinity of −9 kcal/mol, interacted with nine key residues in the active site (Table 1).

These included hydrogen bonds with Lys721, Asp813, and Asp831, hydrophobic interactions with Phe699, Ile735, Leu834, and Lys851, and electrostatic interactions with Lys721, Glu738, Asp831, and Arg817 (Figure 2C,D, Lapatinib), providing a benchmark for comparing cannabinoid derivatives. Importantly, several of these residues, particularly Lys721 and Asp831, are directly involved in ATP coordination and catalytic activity of EGFR. Therefore, similarity in interactions with these residues was considered more relevant than the total number of interacting residues when evaluating cannabinoid binding modes. Among the top five ligands, (1) Cannabinol Heptafluorobutyrate (CID: 91745794) ranked within the best-scoring group (−9.4 kcal/mol) (Table 1). It interacted with eight key residues, forming an electrostatic connection with Asp831, a hydrogen bond with Lys721, and hydrophobic interactions with Phe699, Val702, Lys721, Leu723, Ala 731, Glu734, Ile735 and Glu738 (Figure 2C,D, L1). Notably, Cannabinol Heptafluorobutyrate shares key interactions with Lapatinib, particularly involving Lys721 and Asp831, supporting a similar ATP-competitive binding mode. Its strong binding profile highlights its potential as a high-affinity EGFR inhibitor. (2) 11-Nor-9-Tetrahydro Cannabinol-9-Carboxylate Acyl-D-Glucuronide (CID: 163285414) had a binding affinity of −9.2 kcal/mol (Table 1). It formed hydrophobic contacts with Phe699 and Val702, hydrogen bonds with Glu734 and Glu738, and an electrostatic interaction with Asp831 (Figure 2C,D, L2).

(3) THC-11-Oic Acid Glucuronide (CID: 173519) exhibited a binding affinity of −9.1 kcal/mol and engaged with six residues (Table 1). It established hydrogen bonds with Glu734 and Glu738, hydrophobic interactions with Phe699, Val702, and Lys721, and an electrostatic connection with Asp831 (Figure 2C,D, L3). (4) Cannabinol Pentafluoropropionate (CID: 91745387) showed a binding affinity of −8.5 kcal/mol (Table 1), interacting with seven essential residues, including Phe699, Val702, Ala 731, Glu734, Ile735, Glu738 and Lys851. It formed a wide range of hydrophobic contacts and an electrostatic interaction with Asp831 (Figure 2C,D, L4). (5) 11-Nor-9-Carboxy-Delta9-Tetrahydrocannabinol Glucuronide (CID: 122401304) had a binding affinity of −8.4 kcal/mol and engaged six amino acid residues (Table 1). It formed hydrogen bonds with Glu734, Glu738, and Asp831, hydrophobic contacts with Phe699, Cys773 and Arg817 and an electrostatic interaction with Asp831 (Figure 2C,D, L5). To refine the mechanistic interpretation of the docking results, hydrogen bonding interactions of the control compound and top-ranked cannabinoids were analyzed using Discovery Studio Visualizer.

Specifically, hydrogen bonding interactions were characterized at the atomistic level by explicit identification of hydrogen bond donors (Hd) and acceptors (Ha), together with interaction distances and donor–hydrogen–acceptor (DHA) angles, as detailed below: **Lapatinib**: Ligand(H1, Hd) → Asp813(OD2, Ha) | 2.35 Å | DHA = 154.0°, Ligand(H, Hd) → Asp813(OD2, Ha) | 2.56 Å | DHA = 118.1°, Lys721(CE–H, Hd) → Ligand(F, Ha) | 3.26 Å | DHA = 103.1°; **Ligand 1**: Lys721(CE–H, Hd) → Ligand(F, Ha) | 3.22 Å | DHA = 103.5°; **Ligand 2**: Ligand(H, Hd) → Glu734(OE1, Ha) | 2.17 Å | DHA = 134.8°, Ligand(H, Hd) → Glu738(OE1, Ha) | 2.24 Å | DHA = 168.5°; **Ligand 3**: Ligand(H, Hd) → Glu734(OE1, Ha) | 2.79 Å | DHA = 127.2°, Ligand(H, Hd) → Glu738(OE1, Ha) | 2.90 Å | DHA = 91.3°; **Ligand 4**: -; **Ligand 5**: Ligand(H, Hd) → Glu738(OE1, Ha) | 2.26 Å | DHA = 167.9°, Ligand(H, Hd) → Glu738(OE1, Ha) | 2.68 Å | DHA = 120.1°, Ligand(H, Hd) → Glu734(OE1, Ha) | 2.53 Å | DHA = 114.6°, Ligand(H, Hd) → Asp831(OD1, Ha) | 2.36 Å | DHA = 143.6°.

Lapatinib formed two hydrogen bonds between ligand hydrogen donors and Asp813 (OD2), with distances of 2.35 and 2.56 Å and DHA angles of 154.0° and 118.1°, respectively, along with a weak C–H···F interaction between Lys721 (CE–H, Hd) and a ligand fluorine atom (Ha) at 3.26 Å (DHA = 103.1°), consistent with its ATP-competitive binding mode. Among the cannabinoids, Ligand 1 preserved this interaction pattern through a comparable C–H···F contact with Lys721 (3.22 Å, DHA = 103.5°). Ligand 2 and 3 formed hydrogen bonds with Glu734 (OE1) and Glu738 (OE1), with Ligand 2 showing shorter distances and more favorable angles (2.17–2.24 Å) than Ligand 3 (2.79–2.90 Å). No hydrogen bonds were detected for Ligand 4. In contrast, Ligand 5 exhibited the most extensive hydrogen bonding network, forming multiple hydrogen bonds with Glu734 and Glu738 (2.26–2.68 Å) and a strong interaction with Asp831 (OD1) at 2.36 Å (DHA = 143.6°), a residue critical for ATP coordination. It should be noted that docking scores do not scale directly with the number of hydrogen bonds formed. In AutoDock Vina, the predicted binding affinity reflects a balance between hydrogen bonding, hydrophobic and van der Waals interactions, electrostatic contributions, and penalties associated with ligand desolvation and conformational flexibility. Accordingly, compounds with higher polarity, such as glucuronide-containing ligands, may exhibit lower docking scores despite forming multiple hydrogen bonds.

Across all ligands, residues including Phe699, Lys721, Glu734, Glu738, Asp831, and Ile735 consistently contributed to binding through a combination of hydrogen bonds, hydrophobic contacts, and electrostatic interactions. Notably, Lys721 and Asp831 played pivotal roles due to their involvement in multiple interaction types and their direct relevance to ATP binding and kinase activity. Rather than the total number of interacting residues, engagement of these catalytically relevant residues was considered the primary indicator of inhibitory potential. Several top-ranked cannabinoids reproduced the key interaction pattern observed for Lapatinib, supporting a shared ATP-competitive binding mechanism. These residues have also been highlighted in previous EGFR docking studies [53,54,55], reinforcing their importance in inhibitor design. Based on these interaction profiles, Cannabinol Heptafluorobutyrate, 11-Nor-9-Tetrahydro Cannabinol-9-Carboxylate Acyl-D-Glucuronide, and THC-11-Oic Acid Glucuronide emerged as the best candidates for further investigation. While molecular dynamics simulations were beyond the scope of the present screening-focused study due to computational limitations, they are planned as follow-up analyses for the most promising ligands, together with experimental validation.

#### 3.1.2. Docking Interaction of VEGFR-1 (3HNG) Protein with Cannabinoid Derivatives

**Figure 3:**
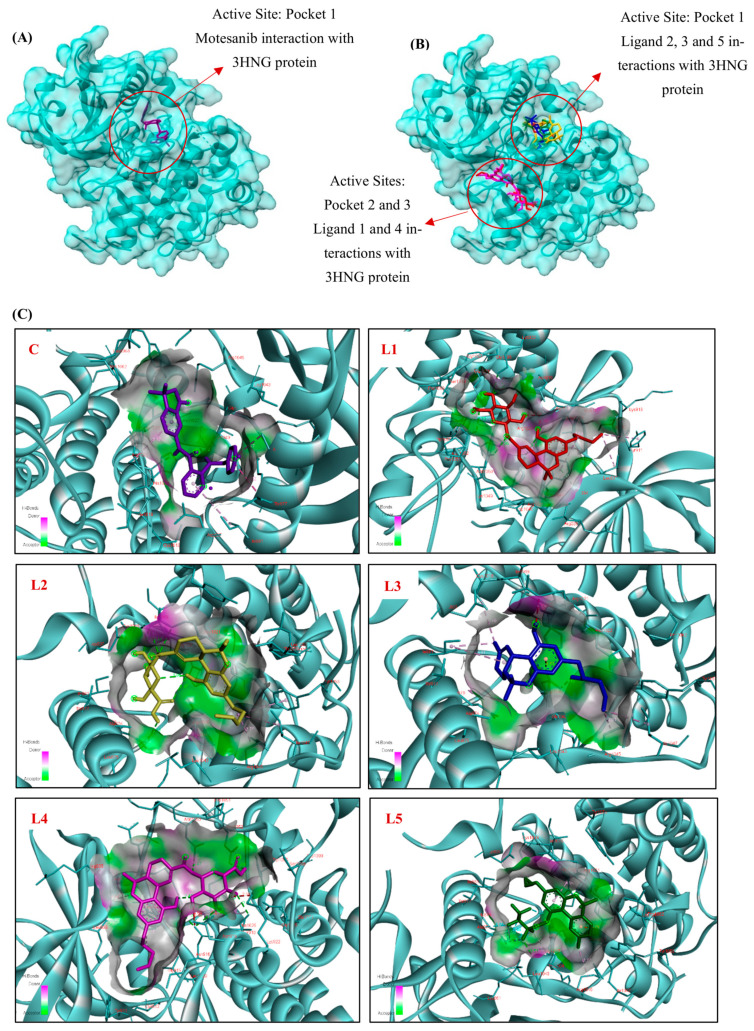

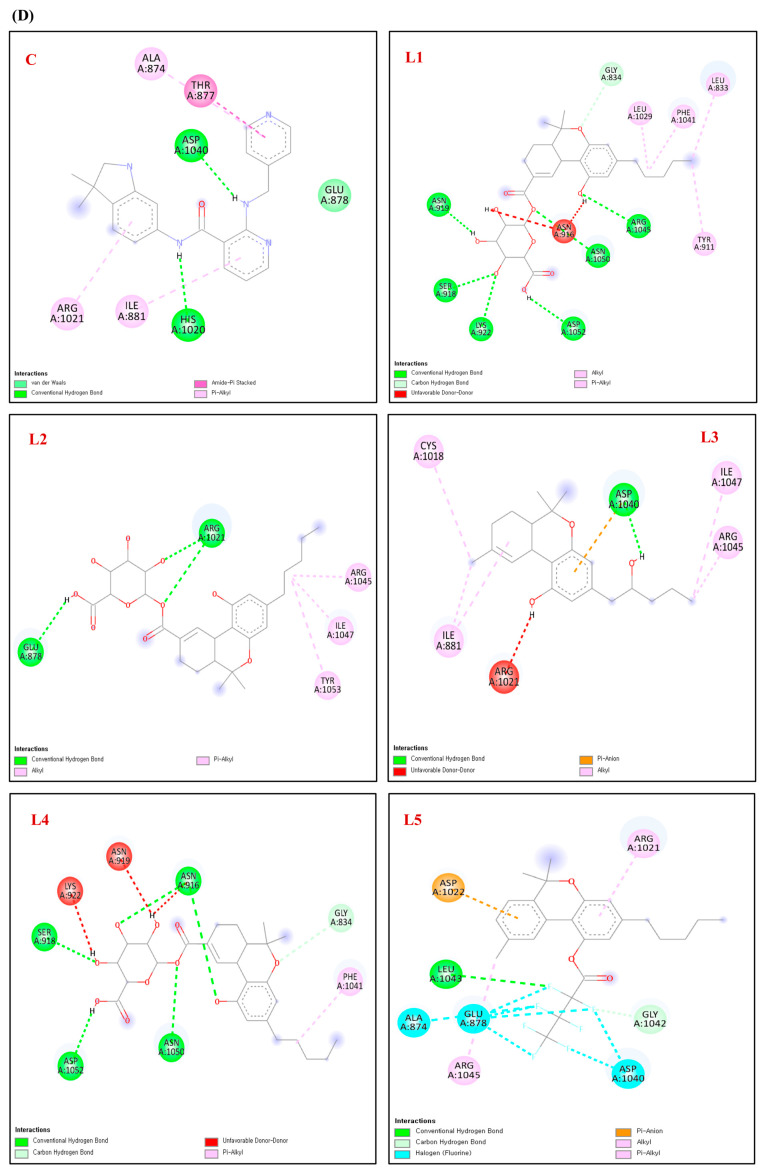
3D Molecular docking simulations of (**A**) Motesanib and (**B**) the top five cannabinoid derivatives at the active site of VEGFR-1 (PDB ID: 3HNG), visualized using UCSF ChimeraX. (**C**) 3D and (**D**) 2D interaction diagrams depicting the binding poses of Motesanib and the top five cannabinoid derivatives with VEGFR-1 (PDB ID: 3HNG), generated using BIOVIA Discovery Studio (C: Control, L1: Ligand 1, L2: Ligand 2, L3: Ligand 3, L4: Ligand 4, L5: Ligand 5). The ligands are color-coded for clarity: purple represents Motesanib (C), red for 11-Nor-9-Tetrahydro Cannabinol-9-Carboxylate Acyl -D-Glucuronide (L1), yellow for 11-Nor-9-Carboxy-Delta9-Tetrahydrocannabinol Glucuronide (L2), blue for 2′-Hydroxy-Delta (9)-THC (L3), pink for THC-11-Oic Acid Glucuronide (L4), and green for Cannabinol, Heptafluorobutyrate (L5).

Blind docking was conducted on VEGFR-1 (PDB ID: 3HNG) to evaluate the interactions of 110 cannabinoid derivatives, using Motesanib as a positive control. Docking was performed using AutoDock Vina in PyRx with a protein-wide blind docking setup. The grid box was centered at (5.54, 18.43, 25.33 Å) with dimensions of 48.88 × 41.89 × 61.36 Å, and the exhaustiveness parameter was set to 8. Docking poses were ranked based on Vina binding affinity scores. Table 2 summarizes the interactions between the top five ligands, ranked based on their binding affinities. Notably, the top five binding results for the cannabinoid derivatives corresponded precisely to the predicted active sites (Figure 1), which also included Motesanib in the control docking (Figure 3A,B). In-depth 2D and 3D representations of the ligand–protein interactions for Motesanib and the top five ligands are shown in Figure 3. The positive control, Motesanib, interacted with six important residues in Pocket 1 and formed hydrogen bonds with His1020 and Asp1040, exhibiting a binding affinity of −8.7 kcal/mol (Table 2).

Motesanib was found to have hydrophobic interactions with Ala874, Thr877, Glu878, Ile881, and Arg1021 (Figure 3C,D, Motesanib), proving to be a potent binder and a standard by which to measure the cannabinoid derivatives. Among the cannabinoid derivatives, (1) 11-Nor-9-Tetrahydro Cannabinol-9-Carboxylate Acyl-D-Glucuronide (CID: 163285414) interacted with two different binding pockets (Pockets 2 and 3) and showed the maximum binding affinity of −8.9 kcal/mol (Table 2). This molecule formed hydrogen bonds with twelve important residues, including Gly834, Ser918, Asn919, Lys922, Arg1045, Asn1050, and Asp1052. Hydrophobic interactions with Leu833, Tyr911, Asn916, Leu1029, and Phe1041 were also noted (Figure 3C,D, L1); these interactions helped to maintain the stability of the ligand–protein complex. (2) 11-Nor-9-Carboxy-Delta9-Tetrahydrocannabinol Glucuronide (CID: 122401304) interacted mostly in Pocket 1 and showed a binding affinity of −8.3 kcal/mol (Table 2). The residues Arg1045, Ile1047, and Tyr1053 showed hydrophobic interactions with the ligand, whereas Glu878 and Arg1021 established hydrogen bonds with it (Figure 3C,D, L2). Five essential residues were implicated in these interactions, which helped to stabilize the ligand inside the active site. (3) 2′-Hydroxy-Delta (9)-THC (CID: 127844) likewise implicated six important residues in Pocket 1 and demonstrated a binding affinity of −8.3 kcal/mol (Table 2).

The ligand established hydrophobic connections with Ile881, Cys1018, Arg1021, Arg1045, and Ile1047, as well as hydrogen bonds with Asp1040 and an electrostatic interaction with Asp1040 (Figure 3C,D, L3). (4) THC-11-Oic Acid Glucuronide (CID: 173519) interacted with eight important residues in Pockets 2 and 3 with a binding affinity of −8.3 kcal/mol (Table 2). While interacting hydrophobically with Phe1041, Asn916, Asn919, and Lys922, it established hydrogen bonds with residues like Gly834, Asn916, Ser918, Asn1050, and Asp1052 (Figure 3C,D, L4). (5) Cannabinol, Heptafluorobutyrate (CID: 91745794) engaged eight important residues in Pocket 1 with a binding affinity of −7.8 kcal/mol (Table 2). In addition to forming hydrophobic connections with Ala874, Glu878, Arg1021, Asp1040, and Arg1045, as well as an electrostatic interaction with Asp1022, the ligand also created hydrogen bonds with Gly1042 and Leu1043 (Figure 3C,D, L5).

Hydrogen bonding interactions were characterized at the atomistic level by explicit identification of Hd and Ha, together with interaction distances and DHA angles, as detailed: **Motesanib**: Ligand(H, Hd) → His1020(O, Ha) | 2.10 Å | DHA = 161.1°, Ligand(H, Hd) → Asp1040(OD1, Ha) | 1.92 Å | DHA = 146.9°; **Ligand 1**: Ligand(H, Hd) → ASN919(OD1, Ha) | 2.31 Å | DHA = 106.9°, Ligand(O, Ha) → Ser918(OG, Hd) | 2.84 Å | DHA = 112.1°, Ligand(O, Ha) → Lys922(NZ, Hd) | 3.02 Å | DHA = 100.2°, Ligand(H, Hd) → Asp1052(OD2, Ha) | 2.72 Å | DHA = 103.3°, Ligand(O, Ha) → Asn1050(ND2, Hd) | 2.95 Å | DHA = 97.9°, Ligand(O, Ha) → Arg1045(NH1, Hd) | 3.11 Å | DHA = 102.4°, Ligand(O, Ha) → Gly834(CA, Hd) | 3.75 Å | DHA = 99.2°; **Ligand 2**: Ligand(H, Hd) → Glu878(OE1, Ha) | 2.72 Å | DHA = 100.3°, Ligand(O, Ha) → Arg1021(NH2, Hd) | 2.94 Å | DHA = 92.9°, Ligand(O, Ha) → Arg1021(NH2, Hd) | 3.10 Å | DHA = 112.6°.

**Ligand 3**: Ligand(H, Hd) → Asp1040(OD2, Ha) | 2.16 Å | DHA = 126.7°; **Ligand 4**: Ligand(O, Ha) → Asn916(ND2, Hd) | 3.08 Å | DHA = 97.5°, Ligand(O, Ha) → Asn916(ND2, Hd) | 3.13 Å | DHA = 119.2°, Ligand(O, Ha) → Asn1050(ND2, Hd) | 2.97 Å | DHA = 95.3°, Ligand(H, Hd) → Asp1052(OD2, Ha) | 2.48 Å | DHA = 103.8°, Ligand(O, Ha) → Ser918(OG, Hd) | 2.80 Å | DHA = 107.6°, Ligand(O, Ha) → Gly834(CA, Hd) | 3.55 Å | DHA = 99.5°; **Ligand 5**: Ligand(F, Ha) → Leu1043(N, Hd) | 3.51 Å | DHA = 125.4°, Ligand(F, Ha) → Gly1042(CA, Hd) | 3.17 Å | DHA = 101.6°.

The positive control, Motesanib, formed two strong hydrogen bonds via ligand hydrogen donors with His1020 and Asp1040, at distances of 2.10 Å (DHA = 161.1°) and 1.92 Å (DHA = 146.9°), respectively, consistent with its established ATP-site binding mode. Among the cannabinoids, Ligand 1 exhibited the most extensive hydrogen bonding network, forming multiple hydrogen bonds with residues spanning Pockets 2 and 3, including Asn919, Ser918, Lys922, Asp1052, Asn1050, and Arg1045, with bond distances ranging from 2.31 to 3.11 Å. Additional weaker interactions with Gly834 were also observed, supporting stabilization across adjacent functional regions of the kinase domain. Ligand 2 primarily interacted within Pocket 1, forming hydrogen bonds with Glu878 and Arg1021 (2.72–3.10 Å), while Ligand 3 established a direct hydrogen bond with the catalytically relevant residue Asp1040 (2.16 Å, DHA = 126.7°), mirroring a key interaction observed for Motesanib. Ligand 4 formed multiple hydrogen bonds involving Asn916, Ser918, Asn1050, and Asp1052, indicating a stable binding mode across Pockets 2 and 3. In contrast, Ligand 5 did not form classical hydrogen bonds but instead engaged in weak C–H/F-mediated contacts with Gly1042 and Leu1043.

Hydrogen bonding interactions were predominantly mediated by residues such as Asp1040, Arg1021, Arg1045, Asn1050, and Asp1052, which are known to play critical roles in ligand recognition and stabilization within the VEGFR-1 kinase domain. Taken together, residues like Asp1040, Arg1021, Arg1045, and Ile1047 continuously facilitated ligand–protein interactions in this study. Furthermore, previous in silico docking analyses of VEGFR-1 have consistently identified residues such as Asp1040, Phe1041, His1020, and Arg1045 as critical for ligand binding and inhibitor design, underscoring their significance [56,57]. The results emphasize the significance of Pocket 1 in ligand stabilization. 11-Nor-9-Tetrahydro Cannabinol-9-Carboxylate Acyl-D-Glucuronide was the top candidate, engaging a high number of pocket residues and yielding a docking score in the same range as the reference inhibitor (Motesanib) and could be considered for further in silico and in vitro evaluation. Importantly, although Pocket 1 corresponds to the canonical ATP-binding site of VEGFR-1, Pockets 2 and 3 are located within structurally and functionally relevant regions of the kinase domain. The observation that several cannabinoids preferentially bind to these pockets suggests alternative but potentially productive interaction sites, rather than nonspecific or non-productive surface binding. Additionally, 2′-Hydroxy-Delta (9)-THC demonstrated good potential as a VEGFR-1 inhibitor, supported by its robust interactions with six key residues in Pocket 1, including a noteworthy electrostatic contact with Asp1040.

#### 3.1.3. Docking Interaction of VEGFR-2 (3U6J) Protein with Cannabinoid Derivatives

**Figure 4:**
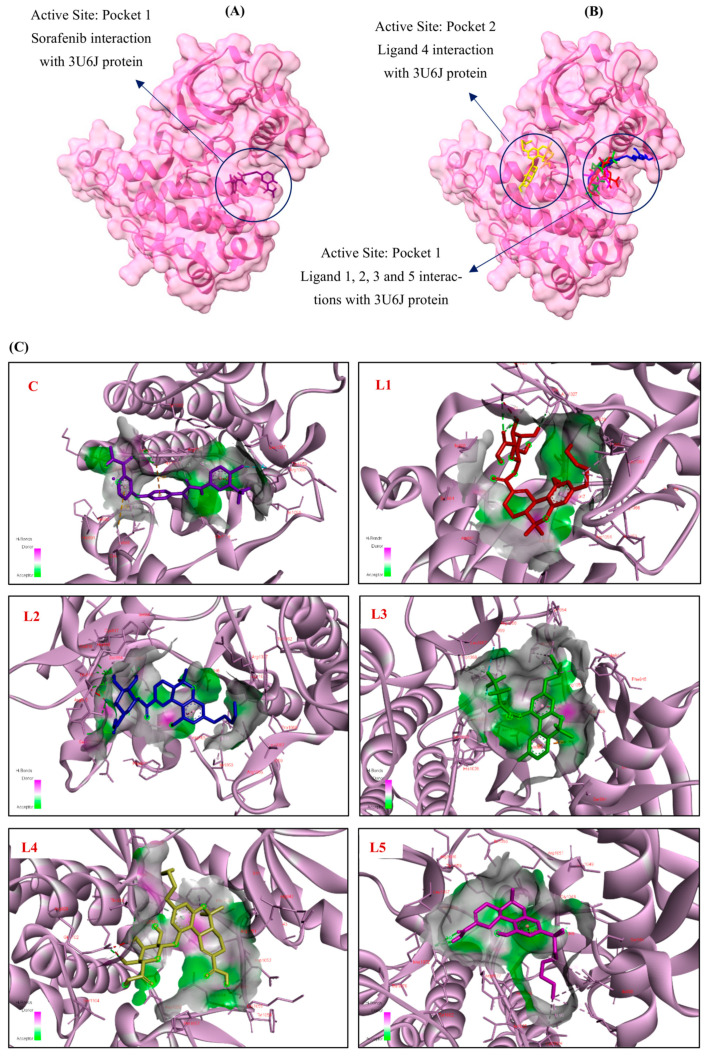

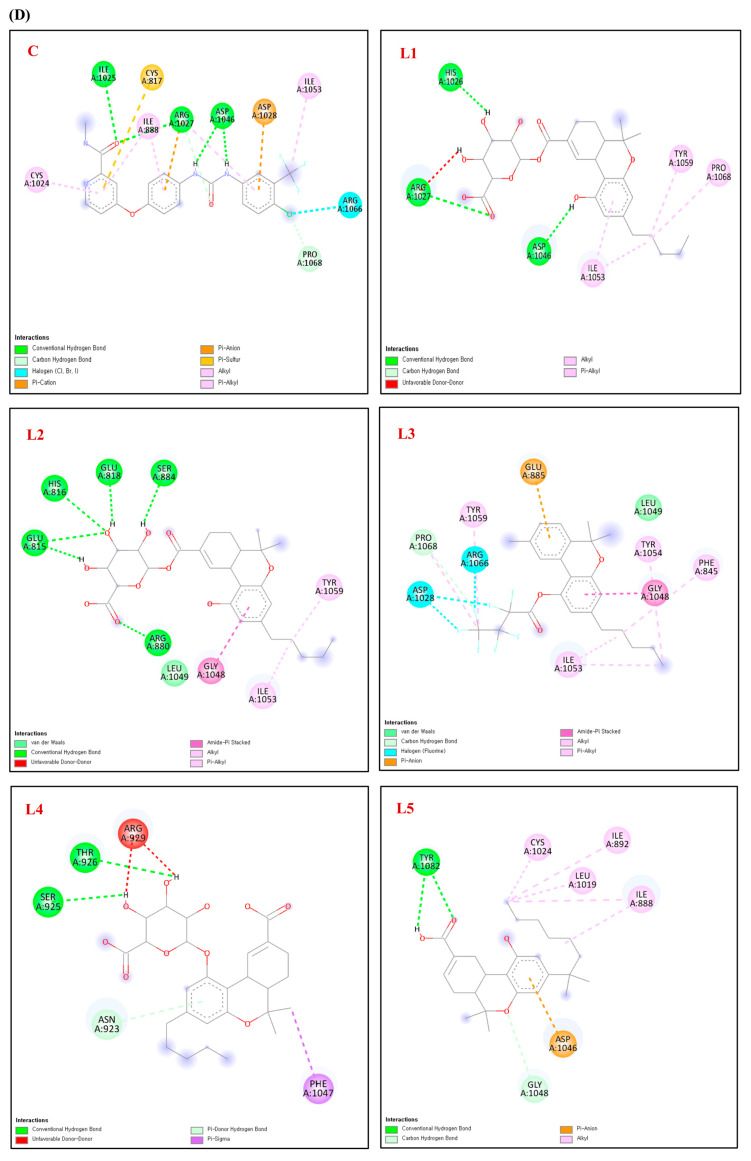
3D Molecular docking simulations of (**A**) Sorafenib and (**B**) the top five cannabinoid derivatives at the active site of VEGFR-2 (PDB ID: 3U6J), visualized using UCSF ChimeraX. (**C**) 3D and (**D**) 2D interaction diagrams depicting the binding poses of Sorafenib and the top five cannabinoid derivatives with VEGFR-2 (PDB ID: 3U6J), generated using BIOVIA Discovery Studio (C: Control, L1: Ligand 1, L2: Ligand 2, L3: Ligand 3, L4: Ligand 4, L5: Ligand 5). The ligands are color-coded for clarity: purple represents Sorafenib (C), red for 11-Nor-9-Tetrahydro Cannabinol-9-Carboxylate Acyl -D-Glucuronide (L1), blue for 11-Nor-9-Carboxy-Delta9-Tetrahydrocannabinol Glucuronide (L2), green for Cannabinol, Heptafluorobutyrate (L3), yellow for 11-9-Tetrahydro Cannabinol-9-Carboxylic Acid -D-Glucuronide (L4) and pink for Ajulemic acid (L5).

The blind molecular docking study investigated the interactions of VEGFR-2 (PDB ID: 3U6J) with 110 cannabinoid derivatives, using Sorafenib as a positive control. Docking was performed using AutoDock Vina in PyRx with a protein-wide blind docking setup. The grid box was centered at (−2.10, −2.91, 18.26 Å) with dimensions of 61.61 × 42.10 × 62.47 Å, and the exhaustiveness parameter was set to 8. Docking poses were ranked based on Vina binding affinity scores. In-depth 2D and 3D representations of the ligand–protein interactions for Sorafenib and the top five ligands are shown in Figure 4. Table 3 summarizes the interaction profiles of the top five ligands, ranked by binding affinity. With a binding affinity of −8.7 kcal/mol, Sorafenib, the positive control, interacted with 9 important residues in Pocket 1 (Table 3). It formed hydrogen bonds with Ile1025, Arg1027, Asp1046, and Pro1068, hydrophobic interactions with Cys817, Ile888, Cys1024, Arg1027, Ile1053 and Arg1066, and electrostatic interactions with Arg1027 and Asp1028 (Figure 4C,D, Sorafenib). These interactions established Sorafenib as a benchmark for evaluating the cannabinoid derivatives.

The top five derivatives of cannabinoids include: (1) 11-Nor-9-Tetrahydro Cannabinol-9-Carboxylate Acyl-D-Glucuronide (CID: 163285414) interacted within Pocket 1 and had a binding affinity of −8.2 kcal/mol (Table 3). It demonstrated its powerful potential as a VEGFR-2 inhibitor by forming hydrogen bonds with His1026, Arg1027, and Asp1046, engaging six important residues, and forming hydrophobic contacts with Arg1027, Ile1053, Tyr1059, and Pro1068 (Figure 4C,D, L1). (2) 11-Nor-9-Carboxy-Delta9-Tetrahydrocannabinol Glucuronide (CID: 122401304) showed a binding affinity of −8.1 kcal/mol (Table 3), engaging three key residues exclusively within Pocket 1. It established hydrophobic contacts with Gly1048, Ile1053, and Tyr1059 and hydrogen bonds with residues such as Glu815, His816, Glu818, Arg880, and Ser884 (Figure 4C,D, L2). (3) Cannabinol, Heptafluorobutyrate (CID: 91745794) interacted with Pocket 1 with a binding affinity of −7.9 kcal/mol (Table 3). In addition to forming hydrophobic contacts with Phe845, Asp1028, Gly1048, Ile1053, Tyr1054, Tyr1059, Arg1066, and Pro1068, it also created hydrogen bonds with Pro1068, as well as an electrostatic interaction with Glu885, implicating seven essential residues (Figure 4C,D, L3). This ligand showed a well-balanced mix of electrostatic and hydrophobic interactions. This ligand demonstrated a balanced combination of hydrophobic and electrostatic interactions. (4) 11-9-Tetrahydro Cannabinol-9-Carboxylic Acid-D-Glucuronide (CID: 163285415) interacted within Pocket 2 with a binding affinity of −7.9 kcal/mol (Table 3). Residues such as Asn923, Ser925, and Thr926 were used to create hydrogen bonds, and two important residues were engaged in hydrophobic interactions with Arg929 and Phe1047 (Figure 4C,D, L4). Its interactions with Pocket 2 indicate a distinct binding orientation despite its moderate binding affinity. (5) Ajulemic Acid (CID: 3083542) likewise showed a binding affinity of −7.9 kcal/mol (Table 3), touching on six important residues in Pocket 1.

Gly1048 and Tyr1082 established hydrogen bonds with it, Ile888, Ile892, Leu1019, and Cys1024 produced hydrophobic connections with it, and Asp1046 formed an electrostatic relationship (Figure 4C,D, L5). To further clarify the binding mechanism, hydrogen bonding interactions were characterized at the atomistic level by explicit identification of Hd and Ha, together with interaction distances and DHA angles, as detailed: **Sorafenib**: Ligand(O, Ha) → Ile1025(N, Hd) | 3.08 Å | DHA = 108.9°, Ligand(O, Ha) → Arg1027(NH1, Hd) | 3.06 Å | DHA = 122.1°, Ligand(H, Hd) → Asp1046(OD1, Ha) | 2.10 Å | DHA = 144.3°, Ligand(H, Hd) → Asp1046(OD1, Ha) | 1.88 Å | DHA = 151.2°, Ligand(O, Ha) → Arg1027(CD, Hd) | 3.53 Å | DHA = 152.7°, Ligand(Cl, Ha) → Pro1068(CD, Hd) | 3.51 Å | DHA = 112.5°; **Ligand 1**: Ligand(H, Hd) → His1026(O, Ha) | 2.28 Å | DHA = 106.7°, Ligand(O, Ha) → Arg1027(NH2, Hd) | 3.02 Å | DHA = 136.8°, Ligand(H, Hd) → Asp1046(OD2, Ha) | 2.35 Å | DHA = 126.4°; **Ligand 2**: Ligand(H, Hd) → Ser884(OG, Ha) | 2.13 Å | DHA = 143.8°, Ligand(H, Hd) → Glu818(OE1, Ha) | 2.33 Å | DHA = 119.5°, Ligand(O, Ha) → His816(N, Hd) | 3.09 Å | DHA = 92.6°, Ligand(O, Ha) → Glu815(N, Hd) | 3.16 Å | DHA = 117.1°, Ligand(H, Hd) → Glu815(OE1, Ha) | 2.91 Å | DHA = 92.5°, Ligand(O, Ha) → Arg880(NH2, Hd) | 3.09 Å | DHA = 109.8°; **Ligand 3**: Ligand(F, Ha) → Pro1068(CD, Hd) | 3.35 Å | DHA = 106.0°; **Ligand 4**: Ligand(H, Hd) → Thr926(OG1, Ha) | 2.77 Å | DHA = 92.1°, Ligand(H, Hd) → Ser925(OG, Ha) | 2.59 Å | DHA = 102.3°, Ligand(π, Ha) → Asn923(ND2, Hd) | 4.03 Å | DHA = 29.8°; **Ligand 5**: Ligand(O, Ha) → Tyr1082(OH, Hd) | 2.90 Å | DHA = 120.0°, Ligand(H, Hd) → Tyr1082(OH, Ha) | 3.00 Å | DHA = 133.1°, Ligand(O, Ha) → Gly1048(CA, Hd) | 3.43 Å | DHA = 107.2°.

The positive control, Sorafenib, formed multiple stabilizing hydrogen bonds within Pocket 1, including two strong interactions with Asp1046 (OD1) at distances of 2.10 and 1.88 Å (DHA = 144.3° and 151.2°), as well as hydrogen bond contacts involving Ile1025 and Arg1027. Additional weak C–H/halogen-mediated interactions with Pro1068 further contributed to ligand stabilization, consistent with its established ATP-competitive binding mode. Among the cannabinoid derivatives, Ligand 1 displayed a binding pattern most closely resembling that of Sorafenib, forming hydrogen bonds with His1026, Arg1027, and Asp1046 (2.28–2.35 Å), thereby engaging catalytically relevant residues within Pocket 1. Ligand 2 formed multiple hydrogen bonds with residues lining the ATP-binding cleft, including Ser884, Glu818, Glu815, His816, and Arg880, indicating a stable yet more polar interaction profile within Pocket 1. Ligand 3 did not establish classical hydrogen bonds but instead exhibited a weak C–H/F-mediated contact with Pro1068, suggesting that hydrophobic and van der Waals contributions dominated its binding mode. Ligand 4 preferentially interacted with Pocket 2, forming hydrogen bonds with Thr926 and Ser925, along with a weaker π-mediated interaction with Asn923, indicating an alternative binding orientation distinct from the canonical ATP site. Ligand 5 formed hydrogen bonds with Tyr1082 and a weak interaction with Gly1048, supporting moderate stabilization within Pocket 1. The docking data underscore the importance of Pocket 1 for VEGFR-2 inhibition, as it served as the primary binding site for most top ligands and the positive control and warrant further in silico and in vitro evaluation. Key residues such as Arg1027, Asp1046, Ile1053, and Pro1068 consistently contributed to ligand stabilization through hydrogen bonds, hydrophobic interactions, and electrostatic forces. According to our findings, several key residues, such as Ile888, Asp1046, Phe1047, His1026, Ile1025, and Arg1045, align with those identified in reliable previous studies on VEGFR-2 inhibition [56,57]. This overlap reinforces the significance of Pocket 1 and its residues in ligand binding and stability, making them key targets for developing potent VEGFR-2 inhibitors.

#### 3.1.4. Cannabinoid Derivatives Commonly Targeting EGFR, VEGFR-1, and VEGFR-2

From the top five cannabinoid derivatives identified for each protein during the docking process, several compounds appeared repeatedly across multiple targets. Consequently, eight distinct cannabinoid derivatives were ultimately identified. Table 4 summarizes these eight candidates, which fall into two primary categories: THC (Tetrahydrocannabinol) derivatives and CBN (Cannabinol) derivatives. The THC derivatives include 11-Nor-9-Carboxy-Delta9-Tetrahydrocannabinol Glucuronide (CID: 122401304), 2′-Hydroxy-Delta (9)-THC (CID: 127844), and THC-11-Oic Acid Glucuronide (CID: 173519). The CBN derivatives include Cannabinol Pentafluoropropionate (CID: 91745387), Cannabinol Heptafluorobutyrate (CID: 91745794), 11-9-Tetrahydro Cannabinol-9-Carboxylic Acid-D-Glucuronide (CID: 163285415), 11-Nor-9-Tetrahydro Cannabinol-9-Carboxylate Acyl-D-Glucuronide (CID: 163285414), and Ajulemic Acid (CID: 3083542). These compounds hold promise for further research into their pharmacological properties as potential inhibitors of EGFR, VEGFR-1, and VEGFR-2, laying the foundation for new cancer therapies (Table 4).

### 3.2. ADME Analysis of Cannabinoid Derivatives

Pharmacokinetic evaluation revealed clear differences among the eight selected cannabinoids (Table 5). Molecular weight is a major determinant of drug absorption, and the optimal range for orally active compounds is typically 150–500 g/mol [48]. Compounds **2** (330.46 g/mol) and **8** (400.55 g/mol) aligned well with this criterion, supporting adequate membrane permeability and systemic uptake. In contrast, Compounds **1**, **3**, **6**, and **7** (~520.57 g/mol) exceeded this range, consistent with their identity as glucuronidated THC metabolites, which are generally more polar and less suitable as oral drug leads due to reduced passive diffusion and increased renal clearance. The remaining two candidates (Compounds **4** and **5**) exhibited extremely high lipophilicity (LogP > 6), which may impair aqueous solubility and limit their bioavailability. Gastrointestinal (GI) absorption patterns were further explained by topological polar surface area (TPSA) values. Glucuronidated metabolites showed high TPSA values (~163 Å^2^), exceeding the threshold typically associated with efficient oral absorption, whereas Compounds **2** and **8** exhibited moderate TPSA values consistent with their higher predicted GI uptake. Accordingly, Compound **2** demonstrated high GI absorption with blood–brain barrier penetration, while Compound **8** combined favorable GI uptake with balanced physicochemical properties. In contrast, glucuronidated compounds (**1**, **3**, **6**, and **7**) exhibited low GI absorption, and the highly lipophilic esters (**4** and **5**) showed poor water solubility, indicating suboptimal profiles for oral administration.

Importantly, these findings indicate that several high-scoring docking hits, particularly glucuronidated metabolites and highly lipophilic esters, are unlikely to be viable orally administered drugs. Instead, such compounds may be more appropriately considered as biochemical probes or metabolic conjugates useful for mechanistic studies rather than conventional drug leads.

**Figure 5:**
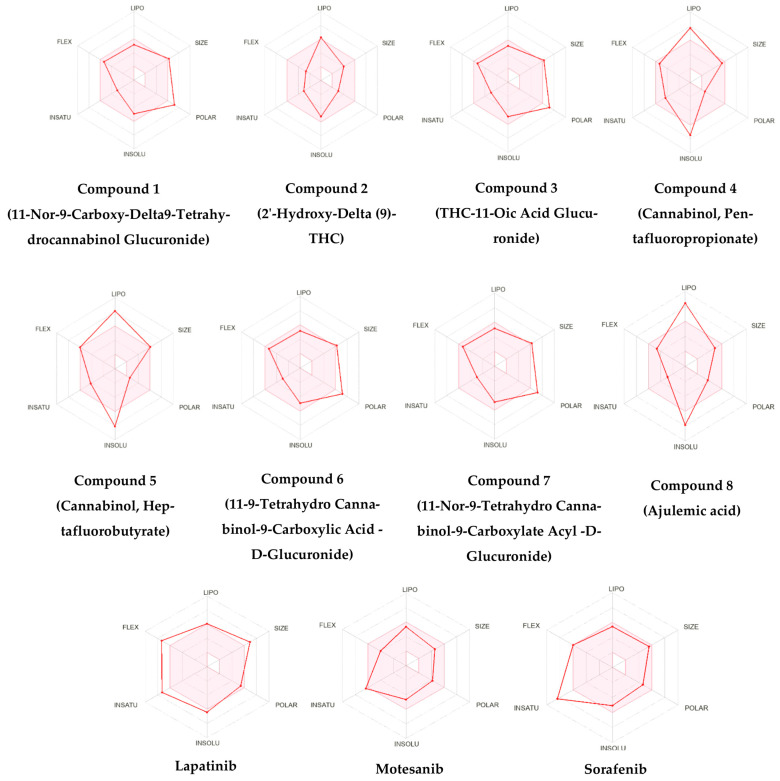
Bioavailability Radar Plot for three positive controls and eight highly effective compounds.

**Figure 6:**
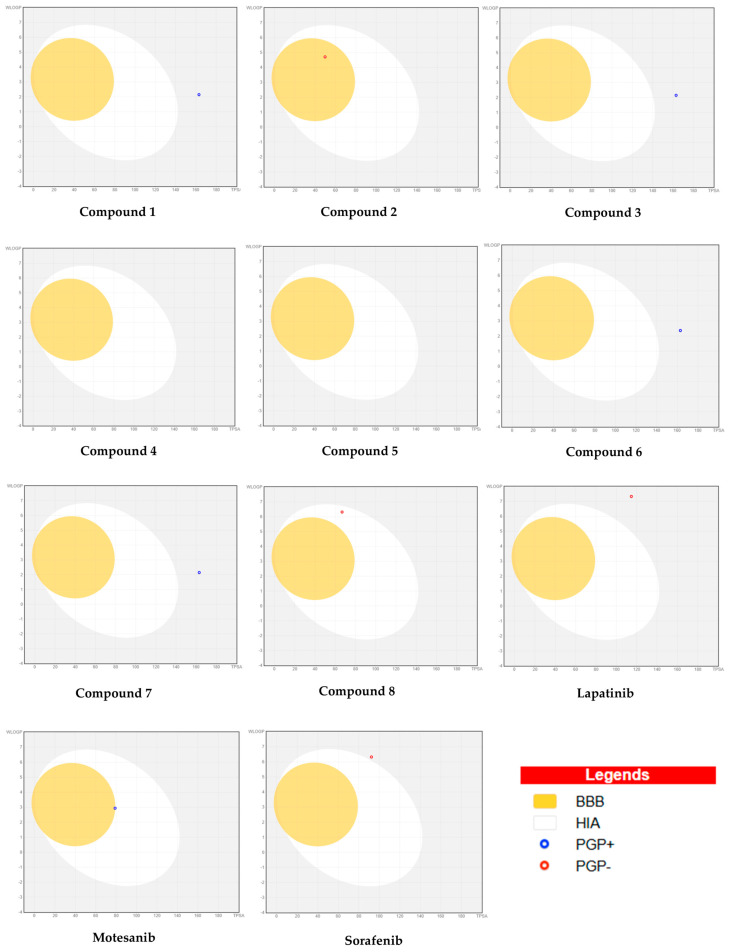
Boiled-Egg model showing predicted gastrointestinal absorption (HIA) and blood–brain barrier (BBB) permeation of the eight selected cannabinoid derivatives and three positive controls. The white area indicates high intestinal absorption and the yellow area indicates BBB penetration. Blue and red dots represent P-glycoprotein substrates (PGP+) and non-substrates (PGP−), respectively.

Overall, 2′-Hydroxy-Delta (9)-THC (Compound **2**) and Ajulemic Acid (Compound **8**) were the only molecules that simultaneously satisfied key drug-likeness parameters—appropriate molecular weight, acceptable lipophilicity, and favorable GI absorption—while maintaining strong multi-target binding. In comparison, THC glucuronides (**1**, **3**, **6**, and **7**) are better interpreted as polar conjugates with limited suitability as primary oral leads, whereas cannabinol esters (**4** and **5**) may require significant optimization or prodrug strategies to overcome solubility constraints. The bioavailability radar plot compares key pharmacokinetic properties, such as lipophilicity, solubility, molecular size, and polarity, for three positive controls and eight effective compounds. Each axis represents a property, with optimal ranges highlighted, allowing a clear visual comparison of drug-like behavior (Figure 5). The Boiled-Egg model summarizes predicted GI absorption and blood–brain barrier permeability for the selected cannabinoids and positive controls (Figure 6).

### 3.3. Toxicity Prediction

The toxicity assessment revealed that all eight cannabinoid candidates were classified within toxicity class 4 according to ProTox-II, corresponding to a low-to-moderate acute toxicity range (LD_50_ = 400–500 mg/kg) (Table 6). However, their absolute LD50 values were lower than those of the reference inhibitors Lapatinib, Motesanib, and Sorafenib (800–1500 mg/kg), indicating a relatively higher predicted acute toxicity for the cannabinoid derivatives. Among the candidates, Compound **2** (2′-Hydroxy-Delta(9)-THC) showed both a lower LD_50_ and a high structural similarity to known toxic compounds, suggesting the need for careful dose optimization in future studies. In contrast, Ajulemic Acid exhibited a more favorable safety profile, with toxicity parameters that were less concerning than those of several other candidates, making it comparatively more suitable for further development. Overall, these results emphasize the importance of experimental validation to refine the therapeutic window, particularly for compounds with lower LD_50_ predictions or structural alerts associated with toxicity.

### 3.4. Structure–Activity Relationship (SAR) Analysis

Based on docking performance across EGFR, VEGFR-1, and VEGFR-2, the recurrent cannabinoid hits cluster into three mechanistically distinct chemotype classes: (i) polar THC glucuronides, (ii) highly lipophilic esterified CBN derivatives, and (iii) moderately polar, drug-like cannabinoids such as 2′-Hydroxy-Delta(9)-THC and Ajulemic Acid. Although these classes differ markedly in physicochemical properties, their docking poses revealed convergent interaction patterns within the kinase ATP-binding pockets. Across all targets, high-affinity ligands consistently relied on a shared pharmacophoric framework, comprising a rigid hydrophobic cannabinoid core that anchors within nonpolar regions of the pocket, complemented by aromatic surfaces and polar functional groups acting as hydrogen bond donors/acceptors or electrostatic anchors. Some docked complexes showed limited unfavorable contacts in the 2D interaction diagrams; however, these were local and outweighed by stabilizing hydrogen-bond and hydrophobic interactions within the ATP-binding pocket. As docking provides a static view of binding, quantitative assessment of such effects would require molecular dynamics simulations, which were beyond the scope of this study. Conserved residues involved in ligand stabilization—such as Lys721 and Asp831 in EGFR, Asp1040 and Arg1045 in VEGFR-1, and Arg1027 and Asp1046 in VEGFR-2—were recurrently engaged, indicating mechanistic coherence despite structural diversity.

Altogether, docking scores were primarily driven by hydrophobic complementarity and contact density, explaining why both glucuronides and highly lipophilic esters achieved favorable binding energies. However, integration with ADME and toxicity predictions highlights clear limitations of these extremes. Glucuronide conjugates, while forming multiple hydrogen bonds, exhibit high molecular weight and polarity, consistent with poor gastrointestinal absorption and rapid elimination. Conversely, highly lipophilic esterified CBN derivatives display excessive logP values, suggesting solubility and bioavailability constraints despite strong hydrophobic binding in silico. In contrast, 2′-Hydroxy-Delta(9)-THC and Ajulemic Acid represent a balanced SAR outcome, combining recurrent multi-target binding with physicochemical properties closer to those of orally active small molecules and comparatively favorable toxicity profiles. Although Ajulemic Acid exhibited a modestly weaker docking score compared to the reference inhibitor, its balanced physicochemical properties, improved ADME predictions, and lower predicted toxicity justified its prioritization over compounds selected solely based on binding affinity. Collectively, this SAR analysis demonstrates that the apparent chemical diversity of the studied cannabinoids masks a shared binding mechanism, while ADME and toxicity filters refine this set toward the most biologically plausible lead scaffolds.

## 4. Discussion

This study evaluated the binding affinities of 110 cannabinoid derivatives to EGFR, VEGFR-1, and VEGFR-2, aiming to identify novel candidates for cannabinoid-based anticancer drugs. Cannabinoids have shown potential in disrupting metabolic pathways like glycolysis, inducing mitochondrial dysfunction, and promoting autophagy and apoptosis, critical mechanisms in tumor suppression. While these findings support their therapeutic potential, additional research is required to elucidate the underlying mechanisms, optimize dosages, establish effective delivery methods, and account for individual variability, as current evidence remains predominantly preclinical [58]. Cannabinoids also offer potential benefits for managing cancer-related symptoms, including pain, anxiety, nausea, depression, and sleep disorders [59]. For instance, THC, a primary cannabinoid, demonstrates analgesic effects comparable to codeine, though its use is limited by side effects such as sedation and mental disturbances [60,61]. Conversely, cannabidiol (CBD), known for its favorable safety profile, has shown significant antiproliferative effects in various cancer cell lines, particularly prostate cancer cells, where it has outperformed other cannabinoids such as cannabichromene and cannabigerol [62]. Clinically, CBD combined with THC has been more effective than THC alone in alleviating cancer pain, especially in cases where opioids fail [63,64]. This suggests that cannabinoid formulations, rather than single-compound therapies, may offer enhanced therapeutic benefits.

EGFR plays a critical role in carcinoma progression, with its overexpression contributing to chemotherapy resistance [65]. From the previous study, molecular docking studies on EGFR (PDB ID: 1M17) have identified promising quinazoline derivatives, which demonstrated superior binding to Erlotinib by interacting with key residues like Met769, Asp831, and Lys721, stabilizing the complex through hydrophobic and electrostatic interactions [45]. Another study on EGFR (PDB ID: 1XKK) highlighted Varlitinib as a highly effective inhibitor, while Imatinib showed moderate efficacy [66]. Additionally, cannabinoids from *C. sativa* were evaluated for EGFR-TKD inhibition (PDB ID: 1M17); Cannabinoids such as Delta9-Tetrahydrocannabinolic Acid (−11.3 kcal/mol) exhibited stronger binding affinities than Tamoxifen (−9.4 kcal/mol) and Erlotinib (−8.2 kcal/mol), while certain terpenes, including Beta-Caryophyllene (−8.4 kcal/mol) showed a docking score comparable to Erlotinib (−8.2 kcal/mol) [53]. These findings emphasize the potential of quinazoline derivatives, cannabinoids, and terpenes as EGFR inhibitors, encouraging further research into dual therapeutic strategies [53,54].

In our investigation, a blind docking study of EGFR (PDB ID: 1M17) was conducted to explore the interactions of 110 cannabinoid derivatives, using Lapatinib as a positive control. Lapatinib, an ATP-competitive inhibitor, exhibited a binding affinity of −9 kcal/mol and interacted with nine key residues through hydrogen bonds (e.g., Lys721 and Asp831), hydrophobic contacts (e.g., Phe699 and Ile735), and electrostatic interactions (Table 1). The top five ligands demonstrated strong binding affinities ranging from −9.4 to −8.4 kcal/mol, aligning with the predicted active site (Table 1, Figure 2). Cannabinol, Heptafluorobutyrate displayed the highest binding affinity (−9.4 kcal/mol), engaging eight key residues, including Lys721 and Asp831. Other notable ligands, such as 11-Nor-9-Tetrahydro Cannabinol-9-Carboxylate Acyl-D-Glucuronide (−9.2 kcal/mol) and THC-11-Oic Acid Glucuronide (−9.1 kcal/mol), also demonstrated stable interactions with crucial residues like Glu734, Glu738, and Asp831 (Table 1, Figure 2). These findings underscore the potential of cannabinoid derivatives as EGFR inhibitors. The consistent involvement of residues such as Lys721 and Asp831 highlights their critical role in ligand stabilization and binding affinity. Importantly, the recurrent involvement of residues such as Lys721, Asp831, and Met769 is not merely descriptive but mechanistically significant. These residues constitute essential elements of the ATP-binding pocket of EGFR and are known to govern ligand orientation, specificity, and inhibitory efficacy through defined noncovalent interactions. Beyond conventional hydrogen bonding and electrostatic contacts, recent studies have highlighted the importance of interaction types such as cation–π recognition and pocket-specific anchoring in achieving selective kinase inhibition [67]. In particular, interactions with positively charged residues such as Lys721 have been shown to stabilize inhibitor positioning within the catalytic cleft, while engagement of acidic residues like Asp831 contributes to conformational restraint and kinase inactivation. The observation that top-ranked cannabinoid derivatives in our study engage these same conserved residues—often through combined hydrogen bonding and hydrophobic complementarity—suggests that their binding is not incidental but follows a recognition pattern analogous to that reported for structurally optimized kinase inhibitors. This residue- and interaction-specific binding mode supports a plausible ATP-competitive mechanism rather than nonspecific surface association, consistent with literature demonstrating that meaningful molecular recognition, rather than binding affinity alone, underlies effective kinase inhibition [68].

The molecular docking of cancer-related proteins provides valuable insights into the design of effective inhibitors by identifying critical interactions at the molecular level. VEGFR-1 (PDB ID: 3HNG), a key target in cancer research, has been extensively studied for its role in tumor angiogenesis. Previous studies utilizing Quantitative Structure–Activity Relationship (QSAR) analysis of VEGFR-1 binding energies for 52 artemisinin-related structures identified essential physicochemical and structural properties necessary for binding. Docking experiments confirmed stable interactions with critical residues, including Asp1040, Cys1039, Val892, Val909, Val841, Lys861, and Glu878. These residues contributed to ligand stabilization across various artemisinin derivatives, such as dimers, dihydroartemisinin-α-hemisuccinate sodium salt, and artesunate, underscoring the utility of QSAR and docking approaches in the design of selective VEGFR-1 inhibitors [69].

Building on these findings, our docking study of VEGFR-1 evaluated 110 cannabinoid derivatives, with Motesanib serving as a positive control. Motesanib exhibited a binding affinity of −8.7 kcal/mol, stabilizing interactions with six residues, including His1020 and Asp1040, within Pocket 1. 11-Nor-9-Tetrahydro Cannabinol-9-Carboxylate Acyl-D-Glucuronide surpassed Motesanib with a binding affinity of −8.9 kcal/mol and engaged 12 residues across Pockets 2 and 3. Other promising cannabinoids, such as 2′-Hydroxy-Delta (9)-THC (−8.3 kcal/mol) and Cannabinol Heptafluorobutyrate (−7.8 kcal/mol), displayed strong binding affinities within Pocket 1 (Table 2, Figure 3). Our findings emphasize the central role of specific residues, particularly Asp1040 and Arg1045, in VEGFR-1 binding, providing a solid framework for providing a framework for prioritizing scaffolds for further computational and experimental testing.

Anti-angiogenic therapy targeting VEGFR-2 has been extensively validated for cancer treatment, with inhibitors such as Sorafenib, Sunitinib, Apatinib, Lenvatinib, Tivozanib, and Pazopanib commonly used against renal cell carcinoma, hepatocellular carcinoma, and leukemia [52,70,71]. VEGFR-2 inhibitors disrupt angiogenic signaling by targeting the ATP-binding pocket within the kinase domain [52,72]. Recent advances identified FO-ARS-123, an artemisinin derivative, as a potent VEGFR-2 inhibitor with nanomolar cytotoxicity against leukemia and breast cancer cells while selectively sparing normal cells [56]. Molecular docking revealed strong binding of FO-ARS-123 at VEGFR-2’s ATP-binding site (PDB ID: 3U6J), involving residues such as Leu840, Val848, Lys868, Ile888, Val899, Thr916, Cys919, Asp1046, and Phe1047, which are frequently highlighted in inhibitor studies for their role in ligand stabilization [56]. Additional pharmacophore modeling and docking studies (PDB ID: 2QU5) identified residues like Val848, Ala866, Lys868, Cys919, and Asp1046 as critical interaction sites [73,74,75]. Among these, Cys919 and Asp1046 consistently emerged as pivotal for ligand binding, underscoring their significance in VEGFR-2 inhibitor design [76,77].

In this study, blind docking of VEGFR-2 (PDB ID: 3U6J) identified promising VEGFR-2 inhibitors among 110 cannabinoid derivatives, using Sorafenib as a positive control. Sorafenib exhibited a binding affinity of −8.7 kcal/mol, interacting with key residues such as Ile1025, Arg1027, Asp1046, and Ile1053 in Pocket 1. The top-performing cannabinoid, 11-Nor-9-Tetrahydro Cannabinol-9-Carboxylate Acyl-D-Glucuronide (−8.2 kcal/mol), engaged six critical residues, including Arg1027, Asp1046, and Ile1053. Cannabinol Heptafluorobutyrate and Ajulemic acid (−7.9 kcal/mol) also showed robust binding within Pocket 1 (Table 3, Figure 4). These results align with previous studies, which identified residues such as Asp1046, Cys919, and Ile1053 as crucial for VEGFR-2 inhibition, further emphasizing the importance of Pocket 1 and its associated residues in ligand design. Similar to EGFR, the interaction of prioritized cannabinoid scaffolds with conserved catalytic residues in VEGFR-1 and VEGFR-2 reflects a recognition-driven binding mechanism, where residue identity and interaction geometry—rather than binding energy alone—govern inhibitory potential, as previously emphasized in kinase-focused molecular interaction studies.

An important aspect of these docking results is that the most favorable binding scores were often associated with the largest and most polar ligands, particularly the THC glucuronides (Compounds **1**, **3**, **6**, and **7**). This is consistent with known limitations of scoring functions such as AutoDock Vina, which tend to favor ligands with larger contact surfaces and a high number of hydrogen bond donors and acceptors. While these metabolites engaged key catalytic residues in the ATP-binding pockets of EGFR, VEGFR-1, and VEGFR-2, their high molecular weight, glucuronic acid conjugation, and predicted low GI absorption suggest that they are more likely to behave as polar clearance metabolites than as optimal oral lead compounds. In contrast, 2′-Hydroxy-Delta (9)-THC and Ajulemic Acid achieved recurrent binding across all three targets with more balanced physicochemical properties, indicating that they may more realistically translate into drug-like scaffolds suitable for further optimization.

The integrated ADME and toxicity analysis further sharpened the distinction between mechanistic hits and realistic lead candidates. In line with recent computational studies on cannabinoid-derived compounds, which have employed integrated docking and ADMET frameworks and reported favorable absorption, distribution, and toxicity profiles—including predicted blood–brain barrier permeability for selected cannabinoids [78]. Ajulemic Acid emerged as the most promising compound, combining an optimal molecular weight, high predicted oral bioavailability, and favorable GI absorption with robust multi-target binding, while maintaining a comparatively acceptable toxicity profile. 2′-Hydroxy-Delta (9)-THC also showed attractive features, including blood–brain barrier penetration, which may be valuable for CNS-involved malignancies, but its lower predicted LD50 and high structural similarity to known toxic compounds indicate a narrower therapeutic window and the need for more cautious dose optimization. By contrast, the THC glucuronides and highly lipophilic cannabinol esters, although strong binders in silico, exhibited suboptimal oral drug-likeness, suggesting that they should be considered as polar metabolites or prodrug-like chemotypes rather than primary oral leads. Taken together, our findings support Ajulemic Acid—and, with appropriate safety considerations, 2′-Hydroxy-Delta (9)-THC—as rational multi-target cannabinoid scaffolds that warrant prioritization in future preclinical studies.

## 5. Conclusions

This study identified eight cannabinoid derivatives showing recurrent binding to EGFR, VEGFR-1, and VEGFR-2, suggesting their capacity to interact with key molecular targets involved in tumor growth and angiogenesis. Molecular docking revealed consistent binding of several cannabinoids to functionally important residues, including Lys721 and Asp831 in EGFR; Asp1040, Arg1021, and Arg1045 in VEGFR-1; and Asp1046, Arg1027, and Ile1053 in VEGFR-2. The predicted binding modes and interaction patterns were comparable to those observed for established reference inhibitors, including Lapatinib, Motesanib, and Sorafenib. When docking results were interpreted in conjunction with ADME and toxicity predictions, Ajulemic Acid and 2′-Hydroxy-Delta (9)-THC emerged as the most realistic lead candidates. These compounds combined consistent multi-target binding with more favorable physicochemical profiles, whereas glucuronidated THC metabolites and highly lipophilic cannabinol esters, despite strong docking scores, displayed suboptimal oral drug-likeness. Overall, Ajulemic Acid—and, with cautious consideration of its predicted toxicity, 2′-Hydroxy-Delta (9)-THC—represent the most promising scaffolds for further investigation. Importantly, the present study is limited to computational observations, and no conclusions regarding biological inhibition or therapeutic efficacy can be drawn without experimental validation. Future studies incorporating molecular dynamics simulations and targeted in vitro assays will be required to validate binding stability and refine the therapeutic potential of these cannabinoid scaffolds.

## Figures and Tables

**Table 1:** Molecular docking results of Lapatinib (positive control) and the top five cannabinoid derivatives interacting with EGFR (PDB ID: 1M17).

No.	Ligand Name	Binding Affinity (kcal/mol)	H-Bond	Hydrophobic Interactions	Electrostatic Interactions	No. of Key Residues in the Active Site
-	Lapatinib (Positive Control)	−9	Lys721, Asp813, Asp831	Phe699, Ile 735, Leu834 and Lys851	Lys721, Glu738, Asp831 and Arg817	9
1	Cannabinol, Heptafluorobutyrate	−9.4	Lys721	Phe699, Val702, Lys721, Leu723, Ala 731, Glu734, Ile735 and Glu738	Asp831	8
2	11-Nor-9-Tetrahydro Cannabinol-9-Carboxylate Acyl -D- Glucuronide	−9.2	Glu734, Glu738	Phe699, Val702	Asp831	5
3	THC-11-Oic Acid Glucuronide	−9.1	Glu734, Glu738	Phe699, Val702, Lys721	Asp831	6
4	Cannabinol, Pentafluoropropionate	−8.5	-	Phe699, Val702, Ala 731, Glu734, Ile735, Glu738 and Lys851	Asp831	7
5	11-Nor-9-Carboxy-Delta9-Tetrahydrocannabinol Glucuronide	−8.4	Glu734, Glu738, Asp831	Phe699, Cys773 Arg817	Asp831	6

**Table 2:** Molecular docking results of Motesanib (positive control) and the top five cannabinoid derivatives interacting with VEGFR-1 (PDB ID: 3HNG).

No.	Ligand Name	Binding Affinity (kcal/mol)	H-Bond	Hydrophobic Interactions	Electrostatic Interactions	No. of Key Residues in the Active Site
-	Motesanib (Positive Control)	−8.7	His1020, Asp1040	Ala874, Glu878, Thr877, Ile881 and Arg1021	-	6 (Pocket 1)
1	11-Nor-9-Tetrahydro Cannabinol-9-Carboxylate Acyl -D-Glucuronide	−8.9	Gly834, Ser918, Asn919, Lys922, Arg1045, Asn1050, Asp1052	Leu833, Tyr911, Asn916, Leu1029 and Phe1041	-	9 (Pocket 2) and 3 (Pocket 3)
2	11-Nor-9-Carboxy-Delta9-Tetrahydrocannabinol Glucuronide	−8.3	Glu878, Arg1021	Arg1045, Ile1047, Tyr1053	-	5 (Pocket 1)
3	2′-Hydroxy-Delta (9)-THC	−8.3	Asp1040	Ile881, Cys1018, Arg1021, Arg1045, Ile1047	Asp1040	6 (Pocket 1)
4	THC-11-Oic Acid Glucuronide	−8.3	Gly834, Asn916, Ser918, Asn1050, Asp1052	Phe1041 Asn916, Asn919 and Lys922	-	5 (Pocket 2) and 3 (Pocket 3)
5	Cannabinol, Heptafluorobutyrate	−7.8	Gly1042, Leu1043	Ala874, Glu878, Arg1021, Asp1040, Arg1045	Asp1022	8 (Pocket 1)

**Table 3:** Molecular docking results of Sorafenib (positive control) and the top five cannabinoid derivatives interacting with VEGFR-2 (PDB ID: 3U6J).

No.	Ligand Name	Binding Affinity (kcal/mol)	H-Bond	Hydrophobic Interactions	Electrostatic Interactions	No. of Key Residues in the Active Site
-	Sorafenib (Positive Control)	−8.7	Ile1025, Arg1027, Asp1046, Pro1068	Cys817, Ile888, Cys1024, Arg1027, Ile1053, Arg1066	Arg1027, Asp1028	9 (Pocket 1)
1	11-Nor-9-Tetrahydro Cannabinol-9-Carboxylate Acyl -D-Glucuronide	−8.2	His1026, Arg1027, Asp1046	Arg1027, Ile1053, Tyr1059, Pro1068	-	6 (Pocket 1)
2	11-Nor-9-Carboxy-Delta9-Tetrahydrocannabinol Glucuronide	−8.1	Glu815, His816, Glu818, Arg880, Ser884	Gly1048, Ile1053, Tyr1059	-	3 (Pocket 1)
3	Cannabinol, Heptafluorobutyrate	−7.9	Pro1068	Phe845, Asp1028, Gly1048, Ile1053, Tyr1054, Tyr1059, Arg1066, Pro1068	Glu885	7 (Pocket 1)
4	11-9-Tetrahydro Cannabinol-9-Carboxylic Acid -D-Glucuronide	−7.9	Asn923, Ser925, Thr926	Arg929, Phe1047	-	2 (Pocket 2)
5	Ajulemic acid	−7.9	Gly1048, Tyr1082	Ile888, Ile892, Leu1019, Cys1024	Asp1046	6 (Pocket 1)

**Table 4:** Overview of the eight cannabinoid derivatives as potential EGFR, VEGFR-1, and VEGFR-2 inhibitors.

Ligand	Name	2D Structure	PubChem ID (CID)	Target (kcal/mol)
Compound **1** (THC derivatives)	11-Nor-9-Carboxy-Delta9-Tetrahydrocannabinol Glucuronide	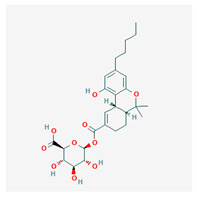	122401304	EGFR (−8.4) VEGFR-1 (−8.3) VEGFR-2 (−8.1)
Compound **2** (THC derivatives)	2′-Hydroxy-Delta (9)-THC	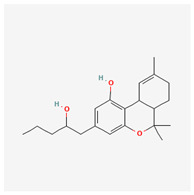	127844	VEGFR-1 (−8.3)
Compound **3** (THC derivatives)	THC-11-Oic Acid Glucuronide	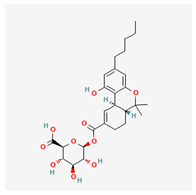	173519	EGFR (−9.1) VEGFR-1 (−8.3)
Compound **4** (CBN derivatives)	Cannabinol, Pentafluoropropionate	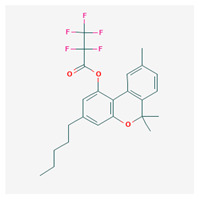	91745387	EGFR (−8.5)
Compound **5** (CBN derivatives)	Cannabinol, Heptafluorobutyrate	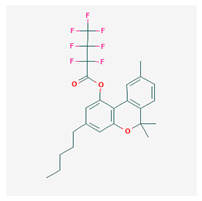	91745794	EGFR (−9.4) VEGFR-1 (−7.8) VEGFR-2 (−7.9)
Compound **6** (CBN derivatives)	11-9-Tetrahydro Cannabinol-9-Carboxylic Acid -D-Glucuronide	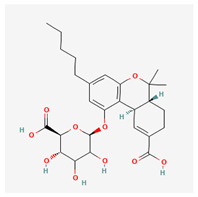	163285415	VEGFR-2 (−7.9)
Compound **7** (CBN derivatives)	11-Nor-9-Tetrahydro Cannabinol-9-Carboxylate Acyl -D-Glucuronide	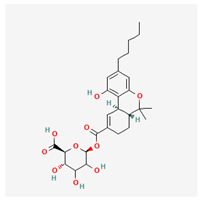	163285414	EGFR (−9.2) VEGFR-1 (−8.9) VEGFR-2 (−8.2)
Compound **8** (CBN derivatives)	Ajulemic acid	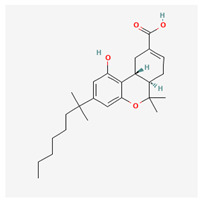	3083542	VEGFR-2 (−7.9)

**Table 5:** Pharmacokinetic (ADME) profiles of eight highly effective compounds identified in this study, generated using the SwissADME server.

	Factor	Molecular Weight (g/mol)	LogP (Consensus LogP)	Solubility (ESOL) Log S	GI Absorption	BBB Penetration	Topological Polar Surface Area (Å^2^)	P-gp Substrate	Bioavailability Score
Compound									
**Compound 1**		520.57	2.45	−4.9	Low	No	162.98	Yes	0.11
**Compound 2**		330.46	4.41	−5.24	High	Yes	49.69	No	0.55
**Compound 3**		520.57	2.35	−4.9	Low	No	162.98	Yes	0.11
**Compound 4**		456.45	6.97	−7.47	Low	No	35.53	Yes	0.55
**Compound 5**		506.45	7.61	−8.11	Low	No	35.53	Yes	0.17
**Compound 6**		520.57	2.26	−4.9	Low	No	162.98	Yes	0.11
**Compound 7**		520.57	2.33	−4.9	Low	No	162.98	Yes	0.11
**Compound 8**		400.55	5.87	−7.87	High	No	66.76	No	0.85
**Lapatinib**		581.06	5.19	−6.44	Low	No	114.73	No	0.55
**Motesanib**		373.45	3.04	−4.67	High	Yes	78.94	Yes	0.55
**Sorafenib**		462.82	4.1	−5.11	Low	No	92.35	No	0.55

**Table 6:** Toxicity prediction of eight highly effective compounds identified in this study, generated using the Protox3 server.

	Factor	Predicted LD_50_ (mg/kg)	Predicted Toxicity Class	Average Similarity (%)	Prediction Accuracy (%)
Compound					
**Compound 1**		500	4	60.37	68.07
**Compound 2**		482	4	96.12	72.9
**Compound 3**		500	4	60.37	68.07
**Compound 4**		400	4	64.29	68.07
**Compound 5**		400	4	60.9	68.07
**Compound 6**		500	4	60.37	68.07
**Compound 7**		500	4	60.37	68.07
**Compound 8**		500	4	79.72	69.26
**Lapatinib**		1500	4	39.97	23
**Motesanib**		850	4	55.78	67.38
**Sorafenib**		800	4	53.45	67.38

## Data Availability

All data generated or analyzed during this study, as well as the web-based tools used, are included in this published article and its Supplementary Materials. The structures of compounds and proteins utilized or analyzed in this study are available in PubChem and the Protein Data Bank (PDB) with accession numbers provided in Supplementary Table S1 and the main text of the manuscript (https://pubchem.ncbi.nlm.nih.gov/ and http://www.rcsb.org).

## Supplementary Materials

The following supporting information can be downloaded at: https://www.mdpi.com/article/10.3390/cimb48020204/s1.

## Abbreviations

Table following abbreviations are used in this manuscript:

EGFR	Epidermal Growth Factor Receptor
VEGFR-1	Vascular Endothelial Growth Factor Receptor 1
VEGFR-2	Vascular Endothelial Growth Factor Receptor 2
ADME	Absorption, Distribution, Metabolism, and Excretion
THC	Tetrahydrocannabinol
THCA	Tetrahydrocannabinolic Acid
CBC	Cannabichromene
CBCA	Cannabichromenic Acid
CBN	Cannabinol
CBD	Cannabidiol
CBDA	Cannabidiolic Acid
CBG	Cannabigerol
CBGA	Cannabigerolic Acid
TKIs	Tyrosine Kinase Inhibitors
PDB	Protein Data Bank
PDBQT	Protein Data Bank Partial Charge and Atom Type
