# Supplementary material for: In Silico Molecular Docking and Pharmacokinetic Evaluation of Cannabinoid Derivatives as Multi-Target Inhibitors for EGFR, VEGFR-1, and VEGFR-2 Proteins
Source: Curr Issues Mol Biol. 2026 Feb 12;48(2):204. doi: 10.3390/cimb48020204 (PMC12939220; doi:10.3390/cimb48020204)
Supplement: Supplementary file 1 [file cimb-48-00204-s001.zip › cimb-4136840-supplementary.pdf]

Supplementary data:

Table S1. Comprehensive library of 110 cannabinoid derivatives evaluated in the study.

Type	No.	Compound	Pubchem ID (CID)	Molecular formula
**Cannabichromene (CBC)**	1	Cannanbichromene	30219	$C_{21}H_{30}O_2$
	2	Nor-Cannabichromene	158435095	$C_{21}H_{28}O_4$
**Cannabichromenic Acid (CBCA)**	3	Cannabichromenic Acid	3084339	$C_{22}H_{30}O_4$
	4	Cannabichromenate	54740355	$C_{22}H_{29}O_4^-$
**Cannabidiol (CBD)**	5	Cannabidiol	644019	$C_{21}H_{30}O_2$
	6	Cannabidiol Hydroxyquinone	11393311	$C_{21}H_{28}O_3$
	7	Cannabidiol-3-Monomethyl Ether	164905	$C_{22}H_{32}O_2$
	8	Cannabidiol Dimethyl Ether	3081957	$C_{23}H_{34}O_2$
	9	(+)-Cannabidiol	36688143	$C_{21}H_{30}O_2$
	10	6beta-Hydroxycannabidiol	53357351	$C_{21}H_{30}O_3$
	11	Abnormal Cannabidiol	89949	$C_{21}H_{30}O_2$
	12	Cannabidiolic Acid	160570	$C_{22}H_{30}O_4$
	13	2-[3-Methyl-6-(Prop-1-En-2-Yl) Cyclohex-2-En-1-Yl]-5-Pentylbenzene-1,3-Diol	521372	$C_{21}H_{30}O_2$
	14	Pre-Cannabidiol Phenethyl Ester	25019755	$C_{30}H_{38}O_4$
	15	Cannabidiol-Aldehyde Diacetate	3044489	$C_{25}H_{32}O_5$
	16	6alpha-HydroxyCannabidiol	53357350	$C_{21}H_{30}O_3$
	17	1''-HydroxyCannabidiol	121596213	$C_{21}H_{30}O_3$
	18	5-(1,1-Dimethylheptyl) Cannabidiol	126670	$C_{25}H_{38}O_2$
	19	7-Hydroxycannabidiol	11301963	$C_{21}H_{30}O_3$
	20	2''-Hydroxycannabidiol	121596214	$C_{21}H_{30}O_3$
	21	4''-HydroxyCannabidiol	53357352	$C_{21}H_{30}O_3$
	22	5''-HydroxyCannabidiol	101621543	$C_{21}H_{30}O_3$
	23	1-Hydroxy-3-N-Pentyl-Cannabidiol	3060519	$C_{21}H_{30}O_2$
	24	Delta6-Cannabidiol	60209648	$C_{21}H_{30}O_2$
	25	1S,2R-Epoxy-Cannabidiol-2',6'-Diacetate	148695	$C_{25}H_{34}O_5$
	26	3''-HydroxyCannabidiol	121596215	$C_{21}H_{30}O_3$
	27	O, O-Dimethyl Cannabidiol	91740340	$C_{23}H_{34}O_2$
	28	7-Carboxycannabidiol	146592489	$C_{21}H_{28}O_4$
	29	Cannabidibutol	59444413	$C_{20}H_{28}O_2$
	30	10-(Diisopropylamino)Cannabidiol Diacetate	24845987	$C_{31}H_{47}NO_4$
	31	10-((4-Aminobutyryl) Amino) Cannabidiol	44149593	$C_{25}H_{38}N_2O_3$
	32	Cannabidiol Ethyl	129210056	$C_{18}H_{24}O_2$
	33	Cannabidiol-D9	71314488	$C_{21}H_{30}O_2$
	34	Abnormal Cannabidiol-D3	71684591	$C_{21}H_{30}O_2$
	35	Cannabidiol Glycolate	86627027	$C_{23}H_{33}O_5^-$
	36	Cannabidiol? -D-Glucuronide	163285562	$C_{27}H_{38}O_8$
	37	Cannabidiol-D9 -D-Glucuronide	163285718	$C_{27}H_{38}O_8$
**Cannabidiolic Acid (CBDA)**	38	Cannabidiolic Acid	160570	$C_{22}H_{30}O_4$
	39	Cannabidiolic Acid, Phenyl-Boronate	91750641	$C_{28}H_{33}BO_4$
	40	Cannabidiolic Acid Methyl Ester	145996527	$C_{23}H_{32}O_4$

**Cannabigerol (CBG)**	41	Cannabigerol	5315659	$C_{21}H_{32}O_2$
	42	Cannabigerol-D9	137699827	$C_{21}H_{32}O_2$
	43	Cannabigerol-C4	131973883	$C_{20}H_{30}O_2$
	44	Cannabigerol (CBG)	160109	$C_{21}H_{32}O_2$
	45	O-Methylcannabigerol	13864080	$C_{22}H_{34}O_2$
	46	Pre-Cannabigerol Phenethyl Ester	25015884	$C_{30}H_{40}O_4$
	47	Cannabigerol Quinone Acid	148268014	$C_{22}H_{30}O_5$
**Cannabigerolic acid (CBGA)**	48	Cannabigerolic Acid	6449999	$C_{22}H_{32}O_4$
	49	Cannabigerolic Acid Monomethyl Ether	24739091	$C_{23}H_{34}O_4$
**Cannabinol (CBN)**	50	Cannabinol	2543	$C_{21}H_{26}O_2$
	51	Cannabinol Methyl Ether	628150	$C_{22}H_{28}O_2$
	52	Dronabinol	16078	$C_{21}H_{30}O_2$
	53	Delta-8-Tetrahydrocannabinol	638026	$C_{21}H_{30}O_2$
	54	Delta.6-Tetrahydrocannabinol	2977	$C_{21}H_{30}O_2$
	55	Delta.1- Tetrahydrocannabinol	2978	$C_{21}H_{30}O_2$
	56	Ajulemic acid	3083542	$C_{25}H_{36}O_4$
	57	8-Hydroxy-Delta (9)-Tetrahydrocannabinol	169652	$C_{21}H_{30}O_3$
	58	Cannabiorcol	59444404	$C_{17}H_{18}O_2$
	59	11-Hydroxytetrahydrocannabinol	644022	$C_{21}H_{30}O_3$
	60	Cannabinol, Pentafluoropropionate	91745387	$C_{24}H_{25}F_5O_3$
	61	Cannabinol, Tetrahydro-	3034462	$C_{21}H_{30}O_2$
	62	Cannabinol-C2	59444399	$C_{18}H_{20}O_2$
	63	Cannabinol, Acetate	90473109	$C_{23}H_{28}O_3$
	64	Cannabinol, Trifluoroacetate	91744577	$C_{23}H_{25}F_3O_3$
	65	Cannabinol, Heptafluorobutyrate	91745794	$C_{25}H_{25}F_7O_3$
	66	Cannabinol-C4	59444392	$C_{20}H_{24}O_2$
	67	Delta4-Isotetrahydrocannabinol	186077	$C_{21}H_{30}O_2$
	68	(-)-11-9-Tetrahydro Cannabinol-9-Methanol-D9	71752421	$C_{21}H_{30}O_3$
	69	11-Nor-9-Tetrahydro Cannabinol-9-Carboxylic-D5 Acid	102320329	$C_{19}H_{24}O_4$
	70	11-Acetoxy-9-Tetrahydro Cannabinol	14263538	$C_{23}H_{32}O_4$
	71	11-9-Tetrahydro Cannabinol-9-Carboxylic Acid-D-Glucuronide	163285415	$C_{27}H_{36}O_{10}$
	72	11-Nor-9-Tetrahydro Cannabinol-9-Carboxylate Acyl -D-Glucuronide	163285414	$C_{27}H_{36}O_{10}$
**Delta-9-tetrahydrocannabinol (Delta-9-THC)**	73	Delta9-Tetrahydrocannabinol; Dronabinol	16078	$C_{21}H_{30}O_2$
	74	Butyl-Delta (9)-Tetrahydrocannabinol	6453891	$C_{20}H_{28}O_2$
	75	8-Hydroxy-Delta (9)-Tetrahydrocannabinol	169652	$C_{21}H_{30}O_3$
	76	1-Nor-9-Carboxy-Delta-9-Tetrahydrocannabinol	108207	$C_{21}H_{28}O4$
	77	11-Hydroxytetrahydrocannabinol	644022	$C_{21}H_{30}O_3$
	78	Delta-9-11-Carboxytetrahydrocannabinol	107885	$C_{21}H_{28}O_4$
	79	1-Trans- Delta.9-Tetrahydrocannabinol	2978	$C_{21}H_{30}O_2$
	80	Tetrahydrocannabinol Acetate	198013	$C_{23}H_{32}O_3$
	81	N-Hexyl-Delta-9-Tetrahydrocannabinol	161906	$C_{22}H_{32}O_2$

	82	O-Methyl-Delta-9 Tetrahydrocannabinol	3082445	$C_{22}H_{32}O_2$
	83	Ethyl-Delta-9-Tetrahydrocannabinol	195772	$C_{18}H_{24}O_2$
	84	11-Nor-Delta (9)-Tetrahydrocannabinol-9-Carboxylic Acid	44814488	$C_{21}H_{28}O_4$
	85	8-Hydroxy-Delta-9-THC	623131	$C_{21}H_{30}O_3$
	86	Delta-9-THC- Trifluoroacetate	530244	$C_{23}H_{29}F_3O_3$
	87	2'-Hydroxy-Delta (9)-THC	127844	$C_{21}H_{30}O_3$
	88	3'-Hydroxy-THC	194062	$C_{21}H_{30}O_3$
	89	6a,7,8,10a-Tetrahydro-1-Hydroxy-6,6-Dimethyl-3-(Pentyl-5,5,5-D3)-6H-Dibenzo (B, D) Pyran-9-Carboxylic Acid, (6aR,10aR)-	76969399	$C_{21}H_{28}O_4$
	90	4'-Hydroxy-THC	6453619	$C_{21}H_{30}O_3$
	91	8,11-Dihydroxy-Delta-9-Tetrahydrocannabinol	126961369	$C_{21}H_{30}O_4$
	92	6-Hydroxymethyl-Delta (9)-Tetrahydrocannabinol	44151059	$C_{21}H_{30}O_3$
	93	Delta-9-Tetrahydrocannabinol Dichloroethyl Carbamoyl Ester	133911	$C_{26}H_{37}Cl_2NO_3$
	94	3',11- Dihydroxy-Delta (9)-Tetrahydrocannabinol	194083	$C_{21}H_{30}O_4$
	95	THC-11-Oic Acid Glucuronide	173519	$C_{27}H_{36}O_{10}$
	96	Methyl 1-Dehydroxy-1-Methoxy-11-Nor-. Delta. -9-Tetrahydrocannabinol-9-Carboxylate	628690	$C_{23}H_{32}O_4$
	97	9-(Hydroxymethyl)-6,6-Dimethyl-3-Pentyl-6a,7,8,10a-Tetrahydrobenzo[C]Chromene-1,8-Diol	6420382	$C_{21}H_{30}O_4$
	98	Methoxy-THC	53802961	$C_{22}H_{32}O_2$
	99	Delta9-Tetrahydrocannabinol Hemisuccinate	162707	$C_{25}H_{34}O_5$
	100	Di-Desoxy-Delta9-Tetrahydrocannabinol	50919315	$C_{21}H_{30}$
	101	5-Desoxy-Delta9-Tetrahydrocannabinol	50919314	$C_{21}H_{30}O$
	102	1-Desoxy-Delta9-Tetrahydrocannabinol	50919316	$C_{21}H_{30}O$
	103	(+)-Delta9-Tetrahydrocannabinol	134740	$C_{21}H_{30}O_2$
	104	8-Oxo-Delta9-Tetrahydrocannabinol	101152951	$C_{21}H_{28}O_3$
	105	11-Nor-9-Carboxy-Delta9-Tetrahydrocannabinol Glucuronide	122401304	$C_{27}H_{36}O_{10}$
	106	8beta,11-Dihydroxy-Delta (9)-THC	148024	$C_{21}H_{30}O_4$
**Delta-9-tetrahydrocannabinolic acid (Delta-9-THCA)**	107	Delta-9-Tetrahydrocannabinolic Acid	98523	$C_{22}H_{30}O_4$
	108	Delta-9- Tetrahydrocannabinolic Acid-C4 B	46889976	$C_{22}H_{30}O_4$
	109	Delta-9-Tetrahydrocannabinolic Acid A	620758	$C_{22}H_{30}O_4$
	110	Delta9-Tetrahydrocannabutolicacid	59444388	$C_{21}H_{28}O_4$

Table S2. Predicted active site amino acids for EGFR (1M17), VEGFR-1 (3HNG), and VEGFR-2 (3U6J) as identified by PrankWeb**.**

Protein	Predicted Active Sites	Chain Name & Residues Number
**EGFR (PDB ID: 1M17)**	Pocket 1	A_694 A_695 A_699 A_702 A_719 A_721 A_723 A_734 A_735 A_738 A_742 A_751 A_766 A_767 A_768 A_769 A_772 A_773 A_813 A_817 A_818 A_820 A_830 A_831 A_833 A_834 A_851 A_853
**VEGFR-1 (PDB ID: 3HNG)**	Pocket 1	A_1013 A_1018 A_1019 A_1020 A_1021 A_1022 A_1038 A_1039 A_1040 A_1042 A_1043 A_1045 A_1047 A_1053 A_807 A_874 A_878 A_881 A_882 A_885 A_891 A_892
	Pocket 2	A_1026 A_1029 A_1039 A_1040 A_1041 A_1044 A_1045 A_1050 A_833 A_834 A_841 A_859 A_861 A_878 A_882 A_892 A_907 A_909 A_910 A_911 A_912 A_913 A_915 A_916 A_919
	Pocket 3	A_1026 A_1052 A_1065 A_1091 A_1098 A_1099 A_918 A_922
	Pocket 4	A_1083 A_1100 A_1118 A_1137
**VEGFR-2 (PDB ID: 3U6J)**	Pocket 1	A_1019 A_1024 A_1025 A_1026 A_1027 A_1028 A_1044 A_1045 A_1046 A_1048 A_1049 A_1053 A_1059 A_1066 A_1067 A_1068 A_868 A_885 A_888 A_889 A_892 A_898 A_899
	Pocket 2	A_1035 A_1045 A_1046 A_1047 A_1050 A_1051 A_840 A_841 A_848 A_866 A_868 A_889 A_899 A_916 A_917 A_918 A_919 A_922 A_923
